# In Silico Discovery of 5′-Modified 7-Deoxy-7-ethynyl-4′-thioadenosine as a HASPIN Inhibitor and Its Synergistic Anticancer Effect with the PLK1 Inhibitor

**DOI:** 10.1021/acscentsci.3c00332

**Published:** 2023-05-11

**Authors:** Eun-Ji Kwon, Karishma K. Mashelkar, Juhee Seo, Yoon-Ze Shin, Kisu Sung, Sung Chul Jang, Sang Won Cheon, Haeseung Lee, Hyuk Woo Lee, Gyudong Kim, Byung Woo Han, Sang Kook Lee, Lak Shin Jeong, Hyuk-Jin Cha

**Affiliations:** † College of Pharmacy, Seoul National University, Seoul 08826, Republic of Korea; § Research Institute of Pharmaceutical Sciences, Seoul National University, Seoul 08826, Republic of Korea; ∥ College of Pharmacy, Pusan National University, Busan 46241, Republic of Korea; ⊥ Research Institute for Drug Development, Pusan National University, Busan 46241, Republic of Korea; # Natural Products Research Institute, Seoul National University, Seoul 08826, Republic of Korea; ∇ Future Medicine Company, Limited, Seongnam, Gyeonggi-do 13449, Republic of Korea; ○ College of Pharmacy, and Research Institute of Drug Development, Chonnam National University, Gwangju 61469, Republic of Korea

## Abstract

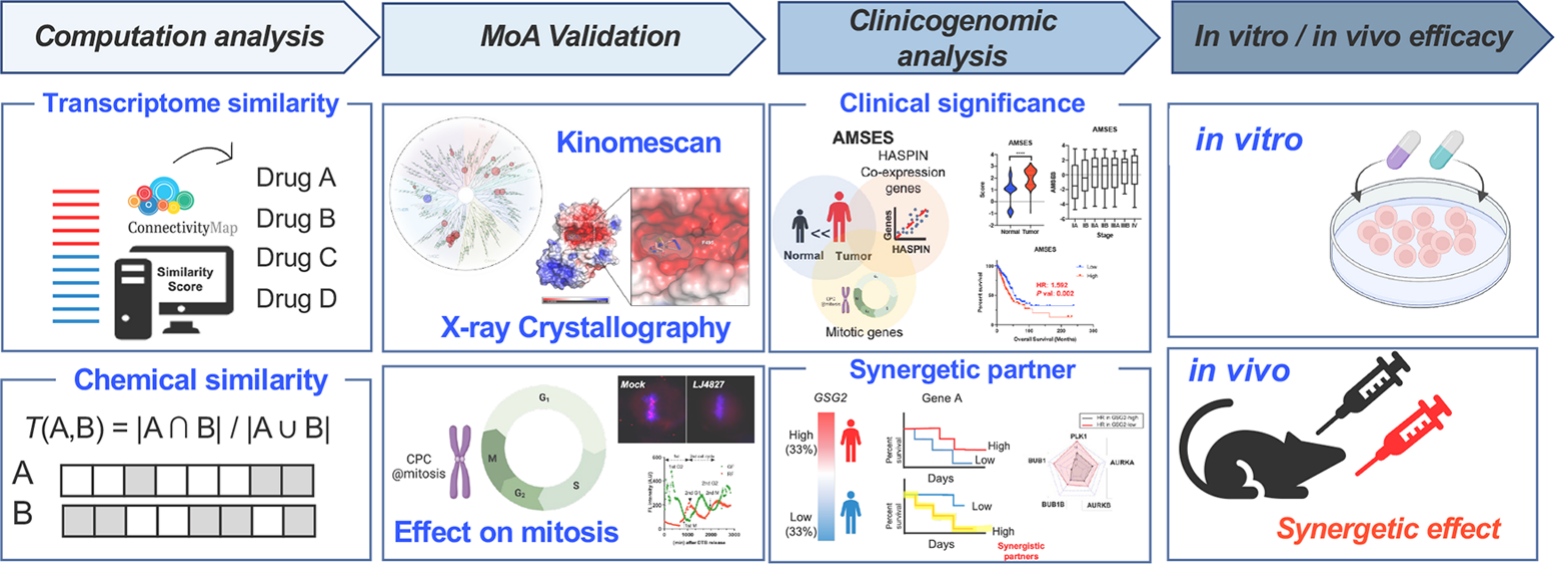

Despite genetic perturbations resulting in embryo lethality for most mitotic kinases, loss of the histone H3 mitotic kinase HASPIN reveals no adverse effect in mice models, establishing HASPIN as a promising target for anticancer therapy. However, developing a HASPIN inhibitor from conventional pharmacophores poses a technical challenge as this atypical kinase shares slight similarities with eukaryotic protein kinases. Chemically modifying a cytotoxic 4′-thioadenosine analogue through high genotoxicity yielded several novel nongenotoxic kinase inhibitors. In silico apporoaches utilizing transcriptomic and chemical similarities with known compounds and KINOMEscan profiles unveiled the HASPIN inhibitor LJ4827. LJ4827’s specificity and potency as a HASPIN inhibitor were verified through in vitro kinase assay and X-ray crystallography. HASPIN inhibition by LJ4827 reduced histone H3 phosphorylation and impeded Aurora B recruitment in cancer cell centromeres but not in noncancer cells. Through transcriptome analysis of lung cancer patients, PLK1 was determined as a druggable synergistic partner to complement HASPIN inhibition. Chemical or genetic PLK1 perturbation with LJ4827 effectuated pronounced lung cancer cytotoxicity in vitro and in vivo. Therefore, LJ4827 is a novel anticancer therapeutic for selectively impeding cancer mitosis through potent HASPIN inhibition, and simultaneous HASPIN and PLK1 interference is a promising therapeutic strategy for lung cancer.

## Abstract 2

Chemically modifying a cytotoxic 4′-thioadenosine analogue through high genotoxicity yielded several novel nongenotoxic kinase inhibitors. In silico apporoaches utilizing transcriptomic and chemical similarities with known compounds and KINOMEscan profiles unveiled the HASPIN inhibitor LJ4827. LJ4827’s specificity and potency as a HASPIN inhibitor were verified through in vitro kinase assay and X-ray crystallography.

## Introduction

Most conventional chemotherapeutics directly or indirectly impede the cell cycle,^1^ triggering severe side effects in actively renewing tissues and repeatedly discouraging clinical results. Thus, the cancer cell cycle must be extensively characterized to identify druggable targets or synthetic lethal partners to disturb cancer cells exclusively.^2^ Haploid germ cell-specific nuclear protein kinase (HASPIN) is encoded by the germ cell-specific gene 2 (*GSG_2_*) and directly phosphorylates histone H3’s threonine 3 residue (H3T3ph) in mitosis.^3^ H3T3ph then serves as a docking site for centromere localization of the chromosome passenger complex (CPC), which strictly controls proper kinetochore-microtubule attachment mediated by Aurora kinase B.^4^ Thus, HASPIN depletion causes chromosome misalignment, premature chromatid separation, and mitotic delay in somatic cancer cell models.^3,4^ Notably, while other prominent mitotic kinase depletions cause severe phenotypic and developmental abnormalities [e.g., CDK1, PLK1, or Aurora A],^6−8^ HASPIN abatement in mice continues to express normal physiology (excluding testicular abnormality),^5^ and HASPIN knockout in embryonic stem cells (ESCs) maintains normal mitosis.^6^ These studies indicate that HASPIN is a promising mitotic target for impeding cancer mitosis exclusively.^7^

Most kinase inhibitors compete with ATP by binding in or around the ATP binding cleft and are classified as type I–IV, depending on the binding mode.^8^ In particular, the conserved ATP/Mg^2+^ binding motif, DFG motif’s Asp(D)-Phe(F)-Gly(G) residues, is responsible for the reversible active or inactive kinase conformation. Thus, type I and II inhibitors, comprising most of the currently known kinase inhibitors, have been designed to lock the ATP pocket conformation as “DFG-in” (type I) or “DFG-out” (type II).^8^ Notably, HASPIN is classified as an atypical eukaryotic kinase (ePK), with Asp-Tyr-Thr (DYT) instead of the DFG motif, and shares low sequence homology with other ePKs.^9^ A few HASPIN inhibitors have been observed with discrete chemical scaffolds (e.g., imidazopyridazine CHR-6494, nucleoside 5ITU, and the β-carboline acridine LDN-211898).^10^ Herein, among cytotoxic 4′-thioadenosine analogue’s chemical derivatives,^11^ the potent HASPIN inhibitor LJ4827 was identified through drug-induced transcriptome computational analysis and subsequent KINOMEscan profiling. Mitotic delay and CPC activity inhibition were cancer cell specific without DNA damage. Moreover, PLK1 was further determined as a synergistic HASPIN inhibition partner based on patient tumor transcriptome and survival data, proposing a novel anticancer therapeutic strategy.

## Results

### Genotoxic-Free 4′-Thioadenosine Analogue MoA Identification

**Scheme 1:**
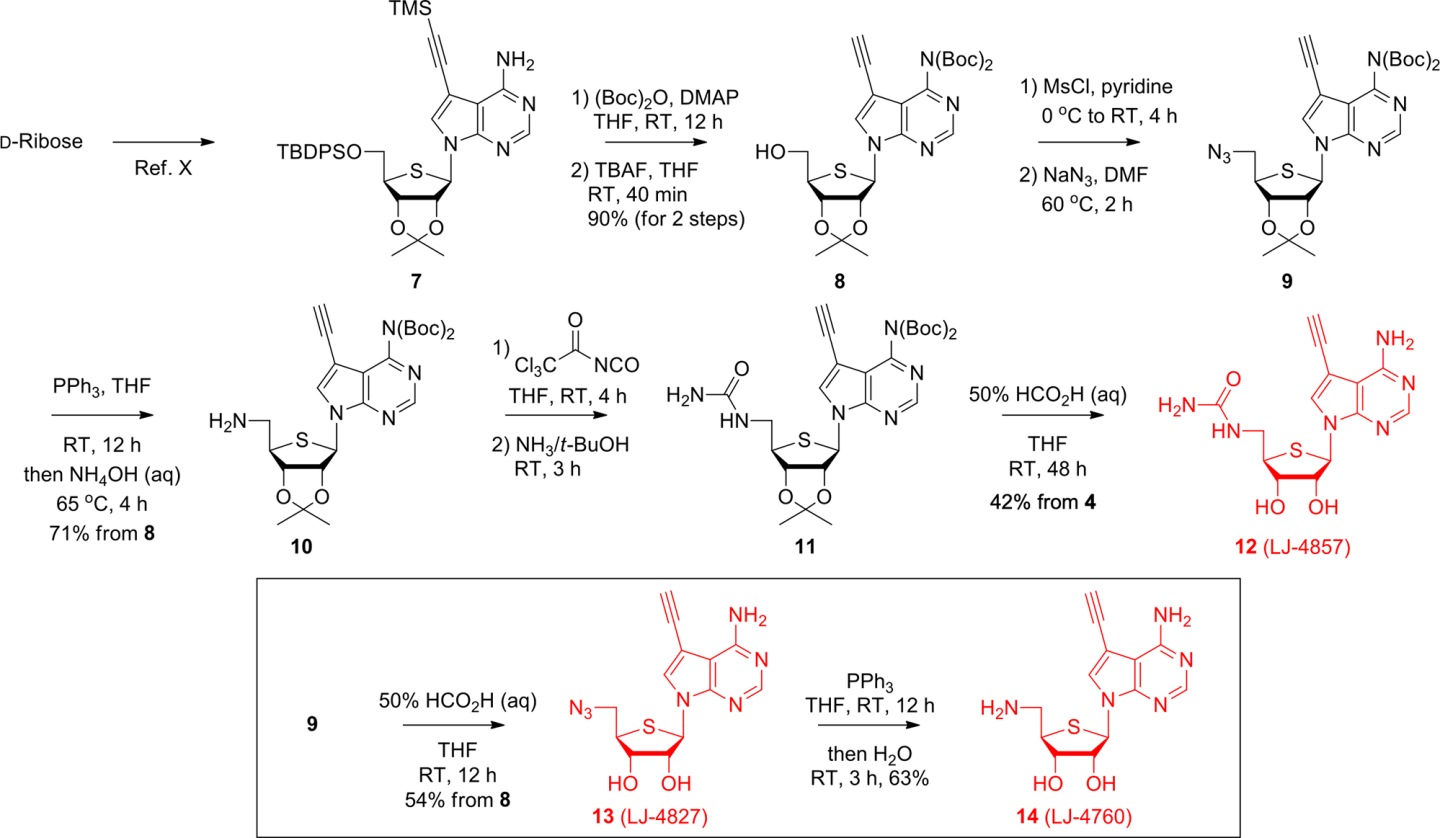
Synthesis of 5′-Modified 7-Deoxy-7-ethynyl-4′-thioadenosine Analogues

**Figure 1:**
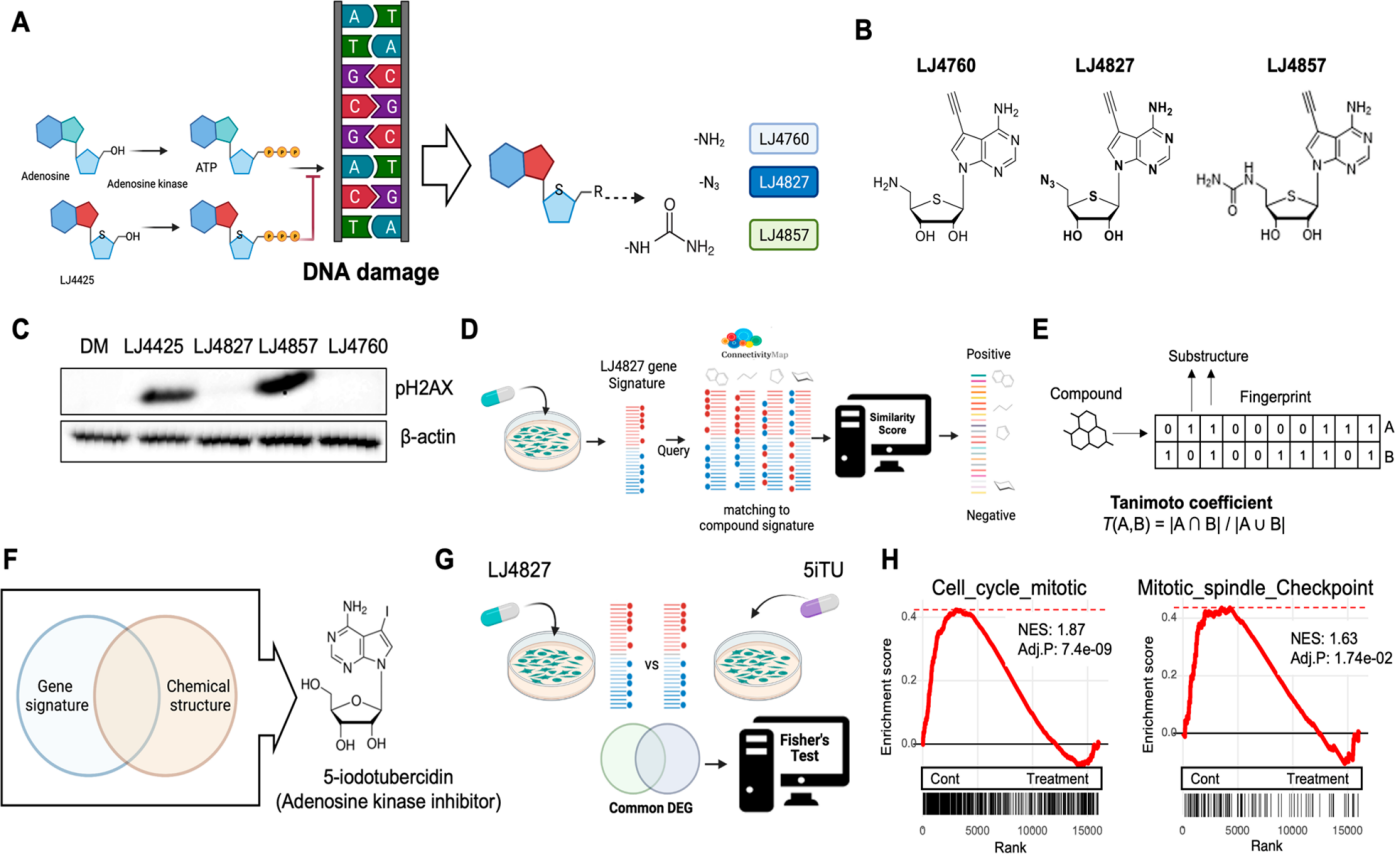
Genotoxic-free 4′-thioadenosine analogue MoA identification. (A) Graphical presentation of adenosine kinase analogue, LJ4425, interfering in DNA elongation due to the existence of a 5′-hydroxyl group. (B) Derivatives of LJ4425 by replacing the 5′-hydroxyl group with amine (LJ4760, left), azide (LJ4827, middle), and urea (LJ4857, right). (C) Immunoblotting analysis for pH2AX on HeLa cells after each derivative’s treatment (500 nM). (D) Scheme of transcriptome-guided MoA inference using the Connectivity Map database. (E) Tanimoto coefficients of compounds predicted in D. (F) Chemical structures of 5-iodotubercidin. (G) Scheme of gene ontology (biological process, BP) analysis of downregulated genes after LJ4827 or 5ITU treatment. (H) GSEA plot of the enrichment of the “Mitotic_spindle_Checkpoint” signature (left) and “Cell_cycle_mitotic” signature (right) in the control group in comparison with the treatment group.

Initially, the cytotoxic multikinase inhibitor LJ4425 with a 4′-thioadenosine structure^11^ was developed as an anticancer drug but was discontinued due to its high toxicity in animal models (data not shown). Similar to other anticancer nucleoside analogues, it was speculated that an LJ4425 5′-hydroxyl group interfering with DNA elongation was responsible for the genotoxicity (Figure 1A) and was replaced to generate multiple 4′-thioadenosine analogues (Scheme 1). For 4′-thionucleoside analogues **6–8** synthesis, d-ribose was converted to the key intermediate **1** through a known method.^11^ Prior to modifying the 5′-hydroxyl group, *N*,*N*-di-Boc protected **1**’s adenine moiety, and the 5′-hydroxyl derivative **2** was produced after removing the TBDPS group. Mesylation of **2** and sodium azide treatment afforded the 5′-azido derivative **3**. Interestingly, a 6-membered 4-azidothiosugar was produced as a minor product under the reaction conditions from a sulfur atom and azide anion interaction that opened the episulfonium ion. This 6-membered 4-azidothiosugar formation can be avoided by employing the Mitsunobu conditions (Ph_3_PN_3_, DIAD, RT, 15 h).^12^ The 5′-azido group of **3** was reduced to the 5′-amino group to produce **4**, which was successively treated with trichloroacetyl isocyanate and methanolic ammonia to induce the 5′-urea derivative **5**. Next, the urea derivative **6** (LJ-4857) was generated by removing **5**’s acetonide protecting group under acidic conditions. Finally, the azide and amine derivatives **7** and **8** were synthesized by hydrolyzing **3** with 50% aq. formic acid to catalyze the azido derivative **7** (LJ-4827), which was first reduced with PPh_3_ and then H_2_O to beget the amino derivative **8** (LJ-4760).

Among the three 4′-thionucleside analogues (Figure 1B), LJ4827 and LJ4760 were not genotoxic based on H2AX phosphorylation levels (at serine139: pH2AX), a marker for double-strand breaks (DSBs)^13^ (Figure 1C). LJ4827 was selected for the MoA study as it has a higher cell-growth impact on various cancer types than LJ4760 (data not shown). Therefore, we first profiled LJ4827-induced differentially expressed genes (DEGs) via RNA sequencing and queried DEGs against the CMap database to search for compounds that exhibited similar expression changes (Figure 1D and Table S1). Among the top-scoring compounds, adenosine kinase (AdK) inhibitors were highly enriched, followed by PKA, IKK, CDK, and JNK inhibitors (Figure S1A). Since chemically similar drugs often share common targets or MoA,^14^ the Tanimoto coefficient determined chemical structural similarities between LJ4827 and the top-scoring compounds (Figure 1E and Table S2). The AdK inhibitor 5-iodotubercidin (5ITU) was most similar to LJ4827 with respect to perturbing effects on the transcriptome and similarity of chemical structure (Figure 1F). However, unexpectedly, AdK inhibition of either 5ITU or LJ4827 was less apparent in vitro under the concentration (Figure S1B) where a distinct anticancer effect was observed (data not shown), implying that LJ4827’s anticancer effect would not incorporate AdK inhibition.

Thus, transcriptomic signatures commonly altered by LJ4827 and 5ITU treatment in cancer cells were examined to identify an anticancer MoA other than AdK (Figure 1G). Ribosome biogenesis and cell cycle-related biological processes were considerably enriched in common DEGs (Figure S1C). In particular, substantially downregulated genes from treatment (e.g., 5ITU or LJ4827) indicated significantly enriched “mitotic spindle checkpoint” and “mitotic cell cycle” genesets compared to the vehicle (Control) (Figure 1H), implying that 5ITU and LJ4827 may affect cancer mitosis similarly. Notably, relative to genes altered by LJ4827 treatment (Figure S1D), 5ITU upregulated genes associated with DNA double-strand break repairs (Figure S1E), which was also revealed by the geneset enrichment analysis (Figure S1F). Corroborating a previous study, 5ITU treatment distinctly produced the pH2AX signal, not LJ4827 (Figure S1G).^15^

### LJ4827 as a Putative HASPIN Inhibitor

**Figure 2:**
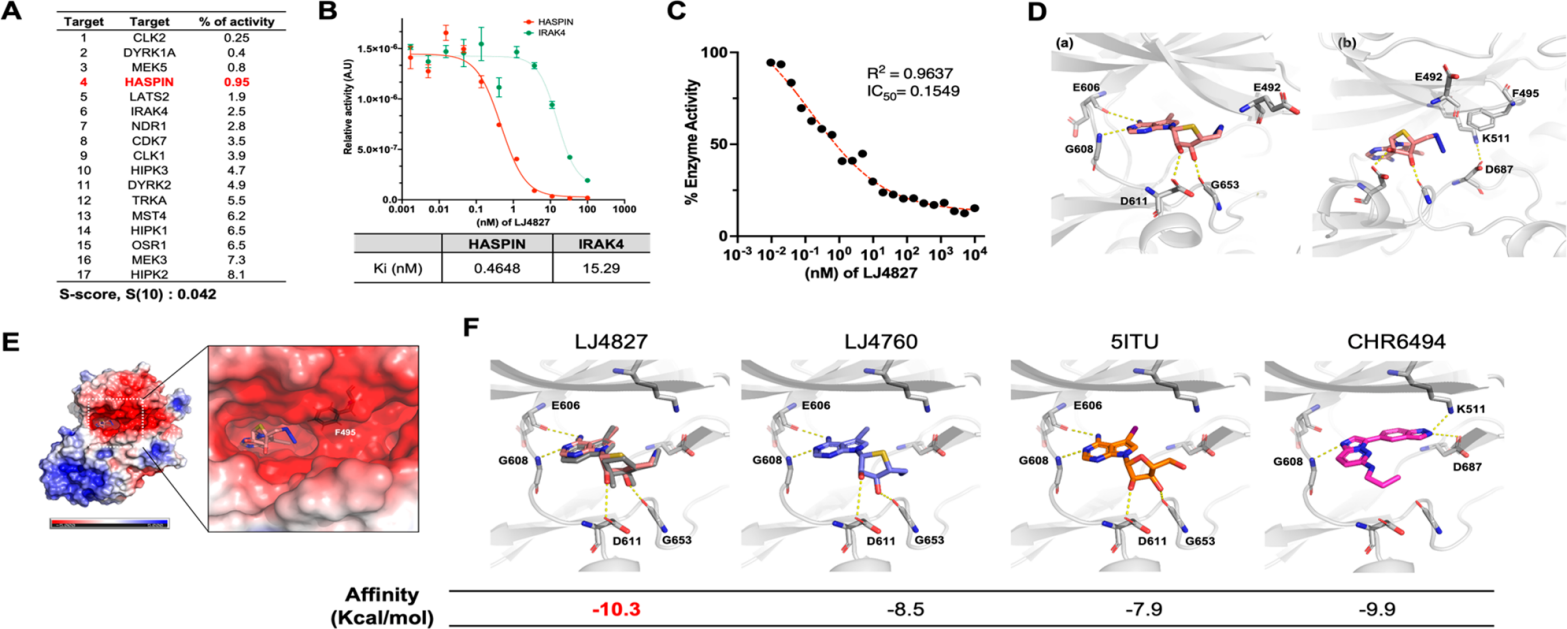
LJ4827 as a putative HASPIN inhibitor. (A) List of hits of 100 nM LJ4827 at selective score 10 [S score, S(10)] = (number of nonmutant kinases with % Ctrl < 10)/(number of nonmutant kinases tested); HASPIN shown in red. (B) Binding constant (*K*_i_) values of LJ4827 and LJ4760 for HASPIN and IRAK4. (C) In vitro kinase assay of HASPIN with the indicated concentrations of LJ4827 using histone H3 as a substrate. (D) Interaction between the HASPIN hinge region and LJ4827 (a) and interaction between HASPIN and the azide moiety of LJ4827 (b). (E) Surface electrostatic potential map of HASPIN in complex with LJ4827. (F) Predicted interaction modes between HASPIN and ligands from docking simulation: (dark gray) LJ4827 from the crystal structure of HASPIN in complex with LJ4827; (salmon) LJ4827 from the control docking experiment; (slate blue) LJ4760; (orange) 5ITU; (magenta) CHR6494. Nitrogen, oxygen, sulfur, and iodine atoms are depicted in blue, red, yellow, and violet, respectively. Values of the binding affinities of indicated compounds on HASPIN from docking simulation.

Next, we conducted a KINOMEscan profiling assay (scanMAX) to determine LJ4827’s affinity for 468 kinases (Figure S2A).^16^ Out of 403 nonmutant kinases, 17 hits were revealed after a 100 nM LJ4827 treatment [i.e., selectivity score, S(10) = 0.042] (Figure 2A). Among these 17 putative target kinases, the mitotic kinase HASPIN that phosphorylates histone H3^3,4^ was included (Figure 2A). Only CLK2, DYRK1A (in the GMGC group), MEK5 (in the STE group), and HASPIN (in the OTHER group) were present at less than 1% of the control cutoff value (Figure S2B). Compared to IRAK4, LJ4827’s *K*_i_ value regarding HASPIN was as low as 0.46 nM (Figure 2B), conveying the greatest affinity to HASPIN among the three 4′-thioadenosine analogues (Figure S2C). For further validation, a HASPIN kinase assay was implemented with the histone H3 peptide, HASPIN’s endogenous substrate, revealing a 0.155 nM IC_50_ of LJ4827 (Figure 2C). Despite similar *K*_i_ values between LJ4827 and LJ4760 (Figure S2C), LJ4827 was more potent for inhibiting HASPIN kinase activity (Figure S2D).

X-ray crystallography at a 2.70 and 2.18 Å resolution determined HASPIN kinase’s crystal structures in a domain complex with LJ4827 or LJ4760 to further verify LJ4827 as a HASPIN inhibitor (Table S3). LJ4827 and LJ4760 were bound in HASPIN’s ATP binding site, and their interaction modes with HASPIN’s hinge region were similar to those of AMP and 5ITU (Figures 2D and S2E).^9^ In addition, within the HASPIN hinge region, *N*^1^ and *N*^6^ of the adenine moiety interacted with Glu606 and Gly608, respectively. The ribose moiety’s 2′- and 3′-hydroxyl groups interacted with Asp611 and Gly653, respectively (Figures 2D and S2E). Interestingly, major structural differences in the LJ4827 (Figure 2E) and LJ4760 (Figure S2F) interaction modes from AMP were observed for the moiety that replaced ribose’s 5′-hydroxyl group. Within HASPIN’s structure complex with AMP, the 5′-phosphate group interacted with Lys511. For 5ITU, the 5′-hydroxyl group forms a hydrogen bond with a bridging water molecule, which subsequently forms a hydrogen bond with Asp687.^9^ In HASPIN’s structure complex with LJ4827, the azide group did not interact with HASPIN.

However, for LJ4760, the amino group interacted with the Glu492’s backbone carbonyl group, inducing Phe495’s phenyl ring to rotate outward, HASPIN’s structure complex with LJ4827, AMP, and 5ITU (Figures 2E and S2F). The APBS-generated electrostatic surface charge demonstrated that compared to that of LJ4827, LJ4760 creates a hole adjacent to HASPIN’s ATP binding region by tilting the phenyl ring outward (Figure 2F).^17^ We speculated that histone H3, HASPIN kinase’s sole endogenous substrate, enters through this hole, potentially accounting for LJ4760’s less potent inhibition than LJ4827 (Figure S2D). Next, we implemented a docking study for LJ4827, LJ4760, 5ITU, and CHR6494 ligands to compare LJ4827’s binding mode with other well-characterized HASPIN inhibitors (e.g., 5ITU^18^ and CHR6494^19^). LJ4827, LJ4760, and 5ITU interactions with HASPIN were the same as those previously mentioned. CHR6494’s imidazopyridine moiety interacted with Gly608, and the indazole moiety interacted with Lys511 and Asp687 (Figure 2F). Of the four ligands tested for docking, LJ4827 expressed the highest affinity (Figure 2F, bottom); Table S3 details these refinement procedures.

### LJ4827’s Cancer-Specific Effects In Vitro and In Vivo

**Figure 3:**
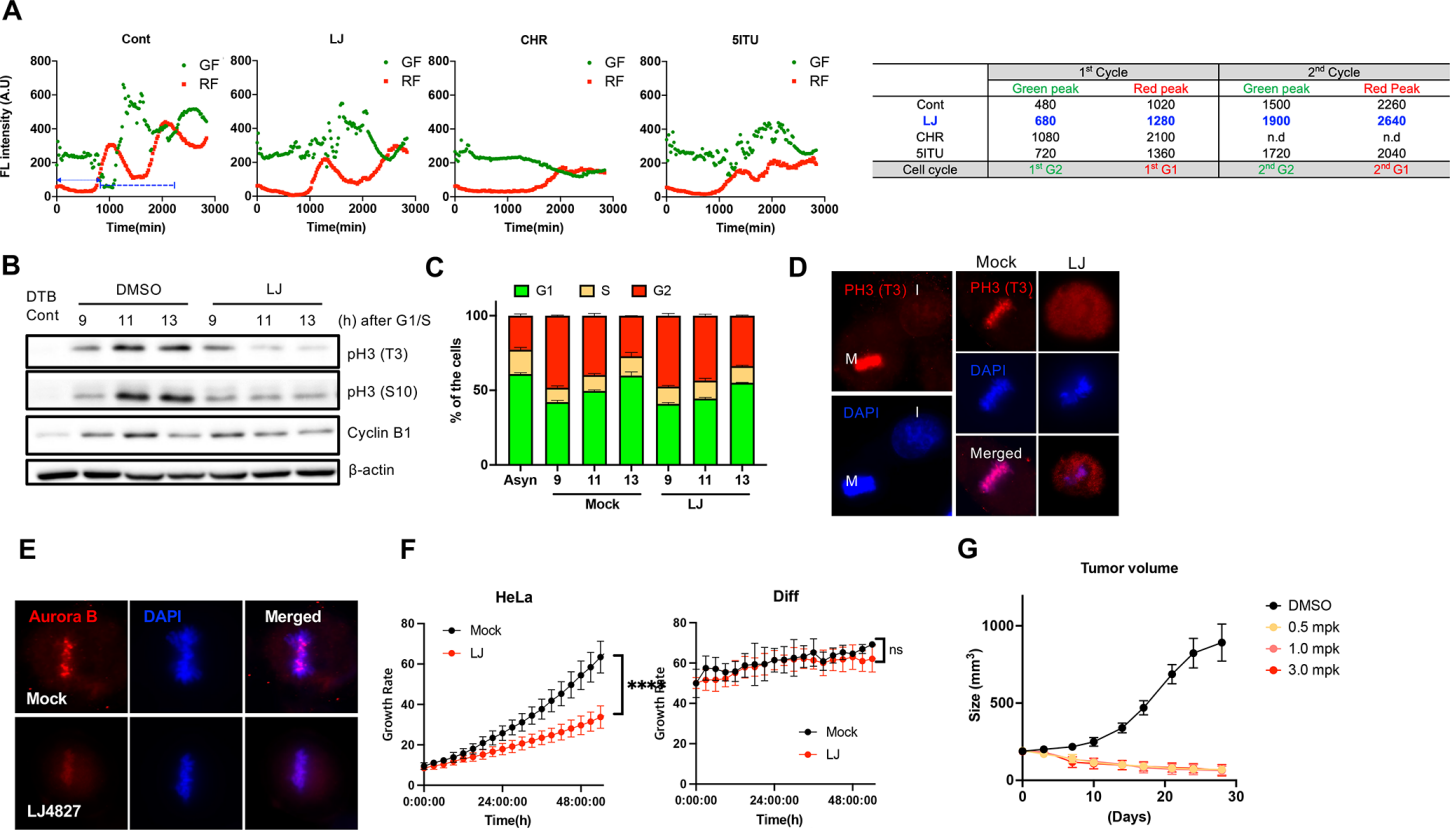
Cancer-specific effect of LJ4827 in vitro and in vivo. (A) Temporal intensity profiles of green (GF) or red fluorescence (RF) from FUCCI-HeLa at indicated time after release from double thymidine block (DTB) with treatment of a 500 nM concentration of each compound: LJ4827 (LJ), CHR6494 (CHR), and 5ITU (top). Summary of time (min, minutes) at the peak of green or red fluorescence intensity after DTB: n.d., not determined (bottom). (B) Immunoblotting analysis for Cyclin B1, phospho-Histone H3 [threonine 3, pH3(T3)], and phospho-Histone H3 [serine 10, pH3(S10)] of A549 at indicated times (h, hours) after release from G1/S (DTB Cont), synchronized by DTB in the absence (DMSO for vehicle) or presence of LJ4827 (500 nM, LJ). (C) Graphical presentation of percent of cell population of G1 (green), S (red), and G2/M (red) at indicated time after G1/S release in the absence (Mock) or presence of LJ4827 (500 nM, LJ). Asyn: Asynchronized control. (D) Fluorescent microscopic images of phospho-Histone H3 [threonine 3, PH3 (T3)] of HeLa at interphase. (E) Immunofluorescent images of Aurora B (red) in HeLa in the absence (Mock) or presence of LJ4827 (500 nM, LJ). (F) Time-dependent proliferation of HeLa and differentiated cells from hESC with or without (Mock) 500 nM LJ4827. (G) Graphical presentation of the tumor volume after injection of indicated dose of LJ4827.

Next, a HeLa-FUCCI system that monitors live cell cycle progression was used to determine the cellular response from LJ4827’s HASPIN inhibition.^20^ As previously described,^21^ green or red fluorescent signal oscillation indicates the first G2 (1st G2), mitosis (1st M), and G1 in the second cell cycle (2nd G1) after release from the G1/S phase, synchronized by a double thymidine block (Figure S3A). In this setting, LJ4827 treatment markedly delayed the first G2 to second G1 timing, similar to the response with 5ITU. In contrast, the cell cycle profile with CHR6494 (CHR) indicated a complete mitotic progression failure, unlike LJ4827 or 5ITU (Figure 3A). Time-lapse images after compound treatment are presented in Figure S3B and Movies S1A–D. According to CHR’s divergent cell cycle profile from LJ4827 or 5ITU (Figure 3A) and the strong pH2AX signal (Figure S3C), CHR’s mitosis entry failure (Figure 3A) results from genotoxic stress responses other than HASPIN inhibition. Notably, a high LJ4827 concentration (i.e., 1.5 μM) completely inhibited mitotic entry, similar to CHR treatment (Figure S3D and Movies S2A–D). LJ4827 usage in subsequent experiments did not surpass 500 nM.

Histone H3 was phosphorylated at threonine 3 [pH3(T3)] for additional validation concerning LJ4827 as a HASPIN inhibitor. LJ4827 significantly weakened HASPIN’s sole known substrate along with phosphorylated histone H3 at serine 10 [pH3(S10)] (Figure 3B), consistent with the cell cycle profile (Figure 3C). Additionally, LJ4827 treatment decreased the pH3(T3) signal associated with the condensed mitotic chromosome (Figure 3D). Moreover, LJ4827 treatment also attenuated Aurora B recruitment at the mitotic chromosome, a representative downstream pH3(T3) event after HASPIN activation (Figure 3E), which was closely associated to the mitotic kinetochore protein CENP-F (Figure S3E).^22^ Notably, normal cells differentiated (Diff) from human embryonic stem cells (hESCs) barely expressed HASPIN, unlike HeLa and undifferentiated hESCs, corresponding to pH3(T3) levels (Figure S3F). LJ4827’s effect on Diff cell growth was negligible, unlike that of HeLa (Figure 3F). LJ4827’s antigrowth effect was more distinct in HeLa than in A549 (Figure S3G), proportionate to the doubling time (DT) and HASPIN expression (Figures S3H and S3I). Similarly, human mesenchymal stem cells (hMSCs), another noncancerous normal cell, retained cell growth (Figure S3J), and Aurora B was localized at the mitotic chromosome upon LJ4827 exposure (Figure S3K). LJ4827’s drastic anticancer effect (Figure 3G) in vivo without visible body weight loss (Figure S3L) verifies that LJ4827 is a safe and potent anticancer molecule.

### In Silico Systematic Approach To Predict a Synergistic Partner of HASPIN Inhibition

**Figure 4:**
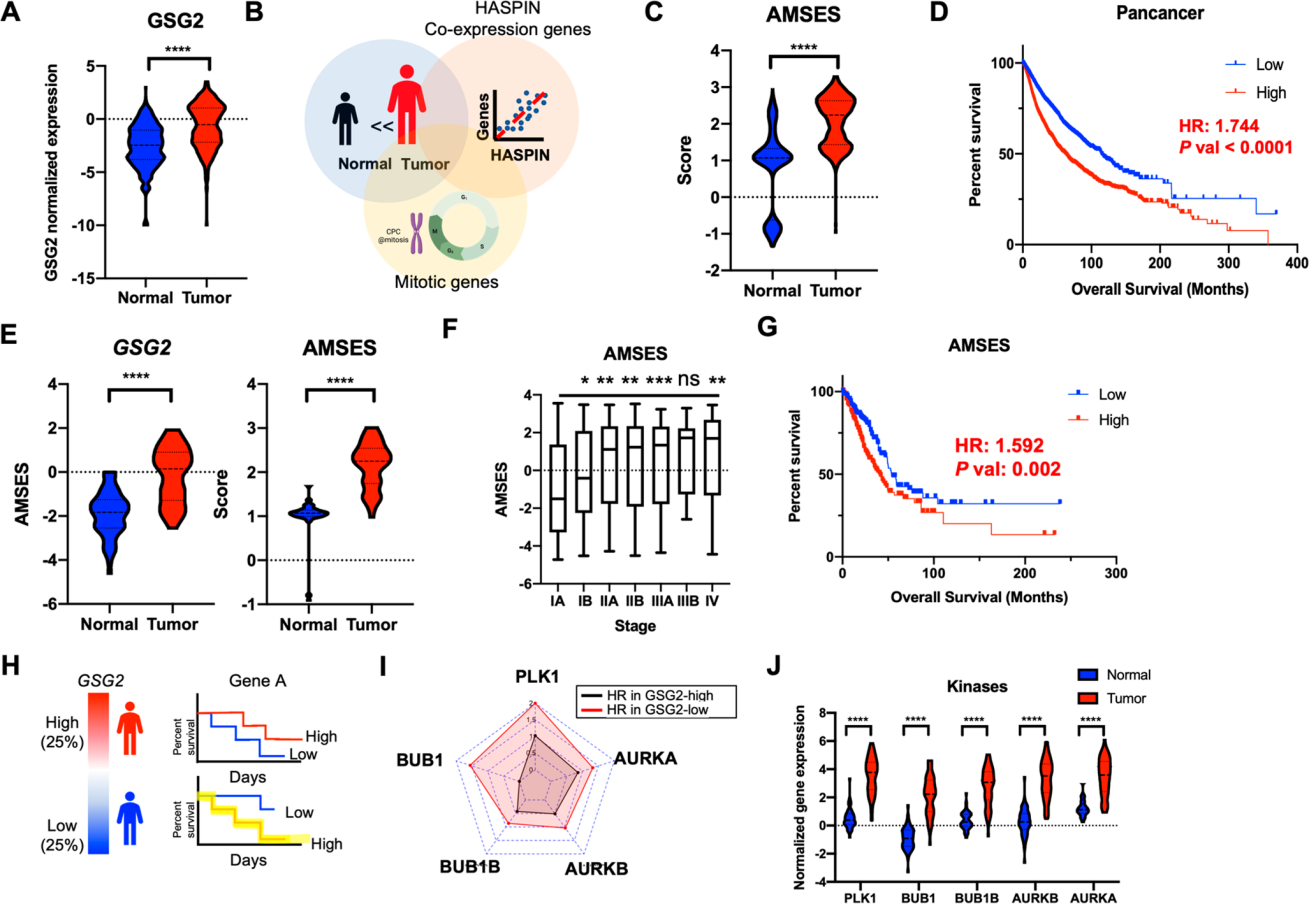
In silico systematic approach to predict a synergistic partner of HASPIN inhibition. (A) Normalized *GSG_2_* expression in normal and tumor pairs (681 patients). (B) Scheme for derivation of the active mitosis signature enrichment score (AMSES). Selection of genes with high expression correlation with GSG_2_ among genes belonging to the “Cell_cycle_mitotic” gene set. (C) AMSES score in normal and tumor pairs of pan-cancer patients (681 patients). (D) Kaplan–Meier survival curves for overall survival with high AMSES in PAN-cancer. (E) Normalized GSG_2_ expression and AMSES score in normal and tumor pairs of LUAD patients (56 patients). (F) AMSES distribution by cancer stage (right). ns: not significant. (G) Kaplan–Meier survival curves of overall survival by AMSES. HR: hazard ratio. (H) Scheme for derivation of synthetic lethal partner based on GSG_2_ expression. (I) Radar plot for hazard ratio (HR) of indicated kinase in GSG_2_-high (black line) or -low (red line) patient group. (J) Normalized gene expression of *BUB1B*, *BUB1*, *PLK1*, *AURKB*, and *AURKA* in normal and tumor groups.

Inspired by the observation that cancer cells exhibit higher *GSG_2_* expression and LJ4827 susceptibility (Figure 3), we further investigated the relationship between *GSG_2_* expression and cancer patient prognosis in the TCGA Pan-Cancer study. Likewise, *GSG_2_* (Figure 4A) and cell cycle-related gene expression levels (Figure S4A and Table S4) were considerably elevated in tumors compared to matched normal samples. Considering HASPIN’s prominence in mitotic progression, we speculated that the high *GSG_2_* expression allows the active mitosis required for cancer’s abnormal cell proliferation. However, *GSG_2_*’s expression level alone would be an insufficient mitotic activity indicator, as *GSG_2_* is continuously expressed throughout the cell cycle.^3^ Therefore, we defined an active mitosis signature as a set of 126 mitotic genes (Table S5) whose expression levels were significantly elevated and correlated with *GSG_2_* expression in pan-cancers. Similar to the mitotic index pathologists use as a prognosis indicator, we leveraged the active mitosis signature to score individual patients regarding enrichment through single-sample GSEA (Figure 4B). The mitosis gene set’s active mitosis signature enrichment score (AMSES) (Table S4) was significantly higher in pan-cancer (Tumor) than the matched normal control (Normal) (Figure 4C) and indicated poor pan-cancer prognosis (Figure 4D). In addition, *GSG_2_* expression and AMSES relevance in lung adenocarcinoma (LUAD) patients were also determined (Figure 4E). Unlike *GSG_2_* expression, AMSES (Figure 4F) corresponded well to LUAD malignancy stages (Figure S4B). Overall survival analysis also substantiated that AMSES (Figure 4G) was more indicative of LUAD prognosis than *GSG_2_* expression (Figure S4C).

An in silico systematic approach identifies synthetic lethal (SL) partner genes of cancer drugs based on a patient’s tumor transcriptome, assuming that the SL partner of a drug is a gene that when coinhibited with the drug target(s) is associated with better prognosis in cancer patients.^23^ Accordingly, we used this approach to identify LJ4827 synergistic partner genes from the TCGA LUAD tumor transcriptome. We hypothesized that HASPIN inhibition was epitomized through low *GSG_2_* expression and sought to locate genes whose low or high expression benefitted patient survival. Given that HASPIN interacts with other kinases during mitosis to regulate chromosome behavior, synergistic partners were preferentially scrutinized within kinase-encoding genes (i.e., readily druggable) in the predefined active mitosis signature. We first divided patients into *GSG_2_*-high and *GSG_2_*-low groups, estimating the association between each kinase’s gene expression and each group’s overall patient survival rate (Figure 4H). Interestingly, *BUB1B*, *BUB*, *AURKB, AURKA*, and *PLK1*, essential kinases that govern mitotic signaling for CPC regulation (Figure S4D), expressed the highest *GSG_2_* expression correlation (Figure S4E). These genes were associated with prognosis in the *GSG2*-low group (hazard ratio, HR > 1) but not in *GSG2*-high (hazard ratio, HR < 1) (Figures 4I, S4F, and S4G) and were significantly higher in tumors than normal cells (Figure 4J). These findings suggest these kinases are putative druggable synergistic partners for HASPIN inhibition with a chemical inhibitor.

### PLK1’s Synergistic Effect with HASPIN Inhibition

**Figure 5:**
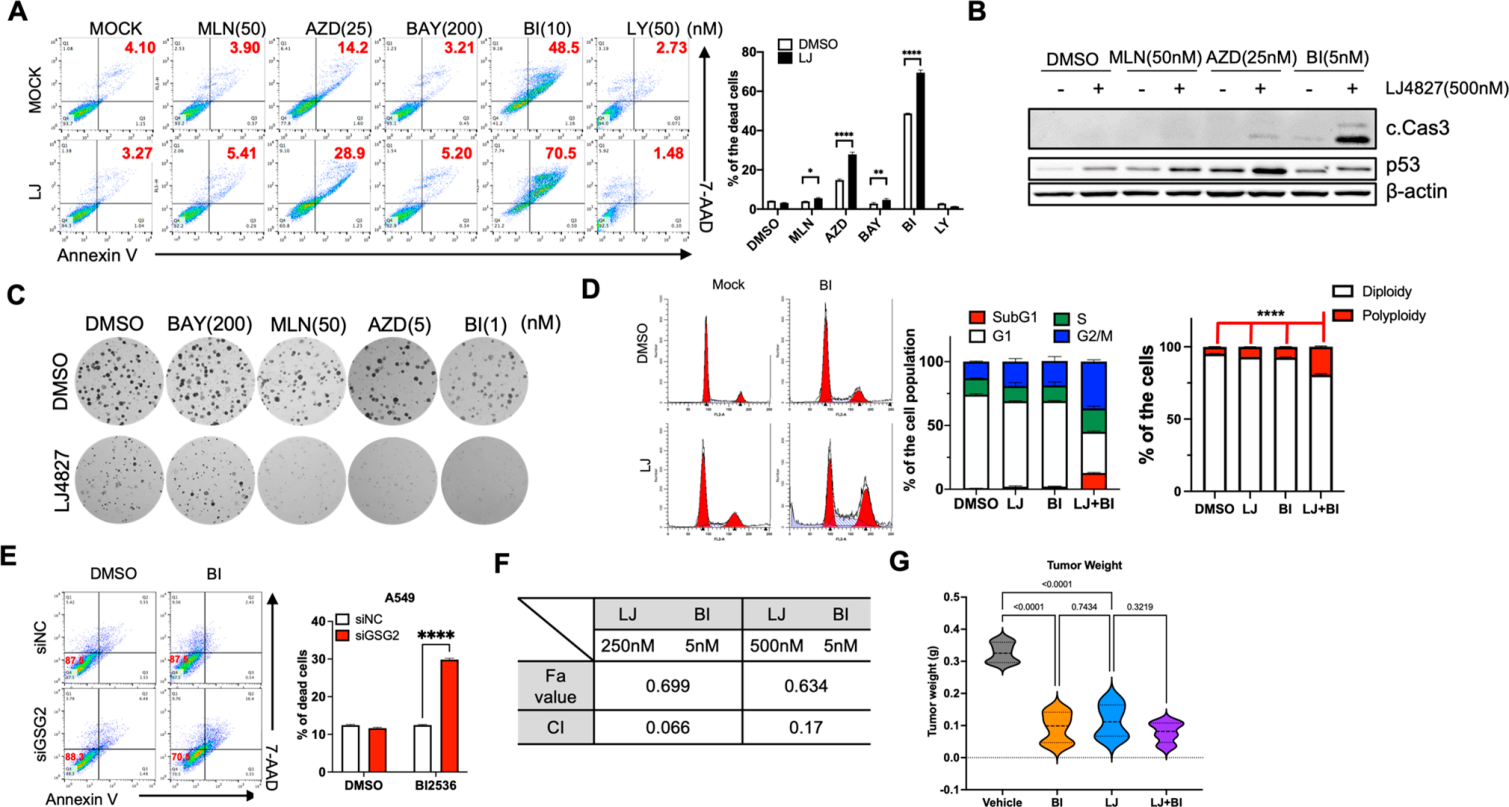
Synergistic effect of PLK1 and HASPIN inhibition. (A) Flow cytometry for Annexin V and 7-AAD 48 h after treatment of indicated dose of inhibitor in A549 cells with 500 nM LJ4827 (left), MLN8237 (MLN, Aurora A/B inhibitor), AZD1152 (AZD, Aurora B inhibitor), BAY1816032 (BAY, BUB1 inhibitor), BI2536 (BI, PLK1 inhibitor), and LY3295668 (LY, Aurora A inhibitor). Cell population of double-positive cells for Annexin V and 7-AAD shown in red. Graphical quantification of dead cells (right). (B) Immunoblotting for cleaved caspase 3 (c.Cas3) and p53 at 24 h after indicated dose of inhibitor with 500 nM LJ4827 in A549 cells. (C) Representative images of clonogenic assay after indicated dose of inhibitor with 500 nM LJ4827 in A549 cells. (D) Cell cycle profile of A549 cells after treatment of BI2536 (BI, 5 nM) in the absence (DMSO) or presence of LJ4827 (LJ, 500 nM) (left). Graphical presentation of the percent of the cells of subG1 (red), G1 (white), S (green), and G2/M (blue) (central). Graphical presentation of the percent of cell population of polyploidy (red) and diploidy (white) in A549 cells at 24 h after indicated treatment (right). (E) Flow cytometry for Annexin V and 7-AAD at 48 h after BI2536 treatment with siRNA (negative control, siNC; GSG_2_, siGSG_2_). Percent of the Annexin V positive population is shown in red. Graphical presentation of dead cells (right). (F) Tables of CI and Fa values of indicated dose of LJ4827 and BI treatment. (G) Graphical presentation of tumor weight from tumor xenografts 30 days after treatment

Next, each kinase’s pharmacological inhibitor was cotreated with LJ4827 to examine synergistic effects. As shown in Figure 5A, adequate cell death occurred through cotreatment with the PLK1 (BI2536: BI) or Aurora B (AZD1152, AZD) inhibitor. Notably, Aurora B was identified as a synergistic HASPIN inhibition partner through genome-wide CRISPR screening,^24^ serving as a positive control for this approach. Despite no apparent cell death from LJ4827 treatment alone (Figure S5A), PLK1 inhibitor’s (BI) strong synergistic effect with LJ4827 treatment on cytotoxicity reached BI’s 5 nM range without any p53 alteration (i.e., no genotoxicity) (Figure 5B). The BI and LJ4827 cotreatment’s antiproliferative effect was evident as low as the 1 nM range (Figure 5C). The cell cycle profile certified that BI and LJ4827 cotreatment markedly increased the Sub-G1 and 4N population, increasing polyploidy (Figure 5D) and suggesting that the proper mitosis failure from simultaneous PLK1 and HASPIN inhibition is closely associated with cell death.

Cell death induction from this cotreatment occurred with a minimum 3 nM BI range (Figure S5B) and 300 nM LJ4827 (Figure S5C). BI’s transient *GSG_2_* depletion of siRNA-sensitized A549 (Figure 5E) and HeLa (Figure S5D) implies that the LJ4827 and BI cotreatment cytotoxicity results from HASPIN perturbation. Thus, the CompuSyn software determined the combination index (CI) value to validate LJ4827’s and BI’s synergism.^25^ A dose–effect curve from single and combined treatments noted cell death measurements from two different LJ4827 (250 and 500 nM) and three BI (1, 3, and 5 nM) doses on A59 cell populations (Figure S5E). The combined LJ4827 and BI synergism (CI < 1) was obtained through the Fa-CI plot from six data points (Figure 5F). Antitumor activity from LJ4827 alone or LJ4827 and BI combined was determined utilizing a nude mouse xenograft model implanted with A549 human lung cancer cells. Compared to the vehicle control group, the tumor volumes in groups treated with LJ4827 or the combination were significantly inhibited without overt toxicity or body weight change (Figure S5F). In addition to the significant antitumor effects from LJ4827 or BI treatment alone, their combined effect was only visible in tumor weight (Figure 5G).

## Discussion

Even after identifying potential compounds with desirable effects, MoA determination is a time-consuming process vital for drug development.^26^ Recently, advanced computational approaches based on large data sets (i.e., chemical and biological data sets) have been proposed to facilitate this step.^27^ Through similarity analysis based on transcriptome profiles (i.e., https://clue.io/) and chemical similarity assays (Figure 1) followed by KINOMEscan profiling of 468 kinases (Figure 2) and X-ray crystallography (Figure 2), we identified HASPIN as a direct target of LJ4827, chemically modified from the genotoxic 4′-thio-adenosine-like multikinase inhibitor. LJ4827’s HASPIN inhibition revealed a clear antimitotic effect on cancer cell lines with high HASPIN expression by interfering with Aurora B recruitment at the mitotic centromere (Figure 3), which would not occur in normal cells. We further improved LJ4827’s anticancer effect by examining potential synergistic partners for cytotoxicity alongside HASPIN inhibition. Rather than genome-wide CRISPR screening, we performed an in silico analysis in which synergistic LJ4827 partners were screened based on LUAD patients’ tumor transcriptomes. Inspired by a previous study,^23^ genes whose coinhibition with HASPIN was associated with a solid prognosis in cancer patients were sought as synergistic partners.

We preferentially examined readily druggable mitotic kinases whose elevated expression represented cancer’s high active mitosis demand. Among five mitotic kinases (BUB1B, BUB, AURKB, PLK1, and AURKA) closely involved in mitotic CPC regulation (Figure 4), PLK1 was selected as a promising synergistic partner of HASPIN expression (Figure 5). Cotreatment of BI2536, a PLK1 inhibitor in phase II clinical trials (NCT00706498 and NCT00710710), and LJ4827 induced distinct cell death at a BI2536 dose as low as 3 nM (Figure S5B). The distinctive synergistic effects of BI2536 and LJ4827 on cell death and mitosis expand potential BI2536 applications, initially a first-generation PLK1 inhibitor that is no longer used in monotherapy.^28^ Due to substantial cell toxicities in actively renewing normal tissues, essential mitotic kinase inhibitors such as PLK1, Aurora A/B, and CDK1 have not been clinically approved despite numerous studies with preclinical promise.^29^ The relatively low normal tissue toxicity of “target therapeutics” results from the high dependency of such “targets” on cancer survival, a mechanism called “oncogenic addiction”.^30^ In contrast, normal cells’ high dependency on these essential mitotic kinases is readily evidenced by severe phenotype effects after knockout,^31^ which may account for the substantial toxicity. In this regard, HASPIN is a promising mitotic target to ensure normal tissue safety, even after complete inhibition, as neither the HASPIN knockout mouse^5^ nor mESCs^6^ exhibit phenotypic abnormality.

## Supporting Information Available

Tables S1–S5: Queried results of CMap with LJ4827 transcriptome, Tanimoto efficient values of Top 18 compounds, data collection and refinement statistics, REACTOME geneset enrichment analysis results in tumor group compared to normal group, and list of active mitosis gene set (XLSX)
Timelapse video of cell cycle after DMSO treatment (MP4)
Timelapse video of cell cycle after LJ4827 treatment (MP4)
Timelapse video of cell cycle after CHR6494 treatment (MP4)
Timelapse video of cell cycle after 5ITU treatment (MP4)
Timelapse video of cell cycle after DMSO treatment (MP4)
Timelapse video of cell cycle after 500nM of LJ4827 treatment (MP4)
Timelapse video of cell cycle after 1μM of LJ4827 treatment (MP4)
Timelapse video of cell cycle after 1.5μM of LJ4827 treatment (MP4)
Experimental details to prepare the chemicals, methods of biological, molecular docking, and computational analysis; frequency of matched compound’s mode of action in query and Tanimoto coefficients of compounds predicted in query, adenosine kinase activity after LJ4827 or 5ITU treatment, gene ontology analysis of downregulated genes by both LJ4827 and 5ITU treatment, gene ontology analysis of downregulated genes by only LJ4827 treatment, gene ontology analysis of downregulated genes by only 5ITU treatment, GSEA plot of the enrichment of the “DNA_double_strand_breeak_repair” signature in the 5ITU-treated group in comparison with the LJ4827-treated group, immunoblotting for pH2AX at 24 h after treatment of LJ or 5ITU in HeLa; graphical presentation of Kinomescan profiling of 100 nM LJ4827 on total 468 kinases, KinMAP of LJ4827 under 1% of control, binding constant values of LJ4827, LJ4857, and LJ4760 for HASPIN, in vitro kinase assay of HASPIN with the indicated concentrations of LJ4827 and LJ4760 using Histone H3 as a substrate, interaction between the HASPIN hinge region and LJ4760, interaction between HASPIN and the amino moiety of LJ4760, surface electrostatic potential map of HASPIN in complex with LJ4760; scheme of the FUCCI system and temporal intensity profiles of green ( or red fluorescence from FUCCI-HeLa at indicated time after release from double thymidine block, time-lapse images of cell cycle after HASPIN inhibitors treatmen, immunoblotting analysis for pH2AX on A549 cells at 24 h after 500 nM treatment of each compound, time-lapse images of cell cycle after LJ4827 concentration-dependent treatment, immunofluorescent images of Aurora B and CENP-F in HeLa, immunofluorescent images of Aurora B in HeLa in the absence or presence of LJ4827, iImmunoblotting analysis for HASPIN and phospho-Histone H3 on HeLa, differentiated cells from hESCs and undifferentiated hESC, relative cell growth rate at 48 h after LJ4827 treatment on A549 and HeLa, relative cell growth rate on HeLa, A549, and differentiated cells from hESCs, immunoblotting for HASPIN in A549, HeLa, and Diff, relative cell growth rate after LJ4827 treatment on HeLa and hMSCs, immunofluorescent images of phospho-Histone H3 Aurora B in hMSCs in the absence or presence of LJ4827, graphical presentation of the body weight after injection of indicated dose of LJ4827, list of enriched REACTOME genesets in tumor group, GSG_2_ expression levels by cancer stage, Kaplan–Meier survival curves of overall survival by GSG_2_ expression, graphical presentation of selected kinases in CPC regulation at the mitotic chromosome, graph of genes highly correlated to GSG_2_ expression, Kaplan–Meier plot for overall survival with BUB1, BUB1B, PLK1, and AURKB expression in GSG_2_ low and high patients, flow cytometry for Annexin V and 7-AAD at 48 h after indicated dose of LJ4827, graphical presentation of dual-positive population with Annexin V and 7-AAD staining, flow cytometry for Annexin V and 7-AAD at 48 h after treatment of indicated dose of BI2536 in the absence or presence of LJ4827, graphical presentation of Annexin V positive cells, flow cytometry for Annexin V and 7-AAD at 48 h after indicated dose of LJ4827 concentration with and without BI2536, quantitative measurement of Annexin V and 7-AAD both negative cells, flow cytometry for Annexin V and 7-AAD at 48 h after BI2536 treatment with siRNA, graphical presentation of Annexin V and 7AAD negative cells, dose– effect curve of six data points of LJ4827 and BI combination, data from CompuSyn software, tumor volume of tumor-bearing mice after the indicated treatment and changes in body weight of tumor-bearing mice after treatment (PDF)

## Abbreviations

HASPIN: haploid germ cell-specific nuclear protein kinase
GSG_2_: germ cell-specific gene 2 protein
CPC: chromosome passenger complex
MoA: mode of action
SMILES: simplified molecular input line entry system
AdK: adenosine kinase
5ITU: 5-iodotubercidin
FUCCI: fluorescence ubiquitination cell cycle indicator
hMSCs: human mesenchymal stem cells
AMSES: active mitosis signature enrichment score
TCGA: The Cancer Genome Atlas
CMap: connectivity map
