# Supplementary material for: In Silico Discovery of 5′-Modified 7-Deoxy-7-ethynyl-4′-thioadenosine as a HASPIN Inhibitor and Its Synergistic Anticancer Effect with the PLK1 Inhibitor
Source: ACS Cent Sci. 2023 May 11;9(6):1140–9. doi: 10.1021/acscentsci.3c00332 (PMC10311661; doi:10.1021/acscentsci.3c00332)
Supplement: Supplementary file 10 — oc3c00332_si_010.pdf [file oc3c00332_si_010.pdf]

Supplementary Information

In Silico Discovery of 5'-Modified 7-Deoxy-7-ethynyl-4'-thioadenosine as a HASPIN Inhibitor and Its Synergistic Anticancer Effect with the PLK1 Inhibitor

Eun-Ji Kwon[1,#], Karishma K. Mashelkar[1,#], Juhee Seo[1], Yoon-Ze Shin[1], Kisu Sung[1], Sung Chul Jang[1,5], Sang Won Cheon[1], Haeseung Lee[3,4], Hyuk Woo Lee[6], Gyudong Kim[7], Byung Woo Han[1,2], Sang Kook Lee[1,5], Lak Shin Jeong[1,2,6*], Hyuk-Jin Cha[1,2*]

[1]College of Pharmacy, Seoul National University, Seoul, Republic of Korea, [2]Research Institute of Pharmaceutical Sciences, Seoul National University, Seoul, Republic of Korea, [3] College of Pharmacy, Pusan National University, Busan, Republic of Korea, [4]Research Institute for Drug Development, Pusan National University, Busan, Republic of Korea, [5]Natural Products Research Institute, Seoul National University, Seoul, Republic of Korea [6]Future Medicine Co., Ltd, Seongnam, Gyeonggi-do, Republic of Korea [7]College of Pharmacy, and Research Institute of Drug Development, Chonnam National University, Republic of Korea

#Equal contribution

*E mail: Hyuk-Jin Cha (hjcha93@snu.ac.kr) and Lak Shin Jeong (lakjeong@snu.ac.kr)

Table of Contents

1. Chemical

1.1 Chemical Synthesis of Final Compounds 4, 5, and 8

2. Biological Assay

3. Molecular Docking Studies

4. Computational Analysis

5. Supplementary Figures (S1~5)

6. Supplementary Tables – Attached Excels

6.1 Table S1. Queried result of CMap with LJ4827 transcriptome

6.2 Table S2. Tanimoto efficient values of Top 18 compounds

6.3 Table S3. Data collection and refinement statistics

6.4 Table S4. REACTOME geneset enrichment analysis result in tumor group compared to normal group

6.5 Table S5. List of active mitosis gene set

7. Movies -Attached Videos

7.1 Movie S1.

(a) Timelapse video of cell cycle after DMSO treatment

(b) Timelapse video of cell cycle after LJ4827 treatment

(c) Timelapse video of cell cycle after CHR6494 treatment

(d) Timelapse video of cell cycle after 5ITU treatment

7.2 Movie S2.

(a) Timelapse video of cell cycle after DMSO treatment

(b) Timelapse video of cell cycle after 500nM of LJ4827 treatment

(c) Timelapse video of cell cycle after 1μM of LJ4827 treatment

(d) Timelapse video of cell cycle after 1.5μM of LJ4827 treatment

8. References

1. Chemical

Chemical Synthesis of Final Compounds 4, 5, and 8 (Scheme S1)

Scheme S1

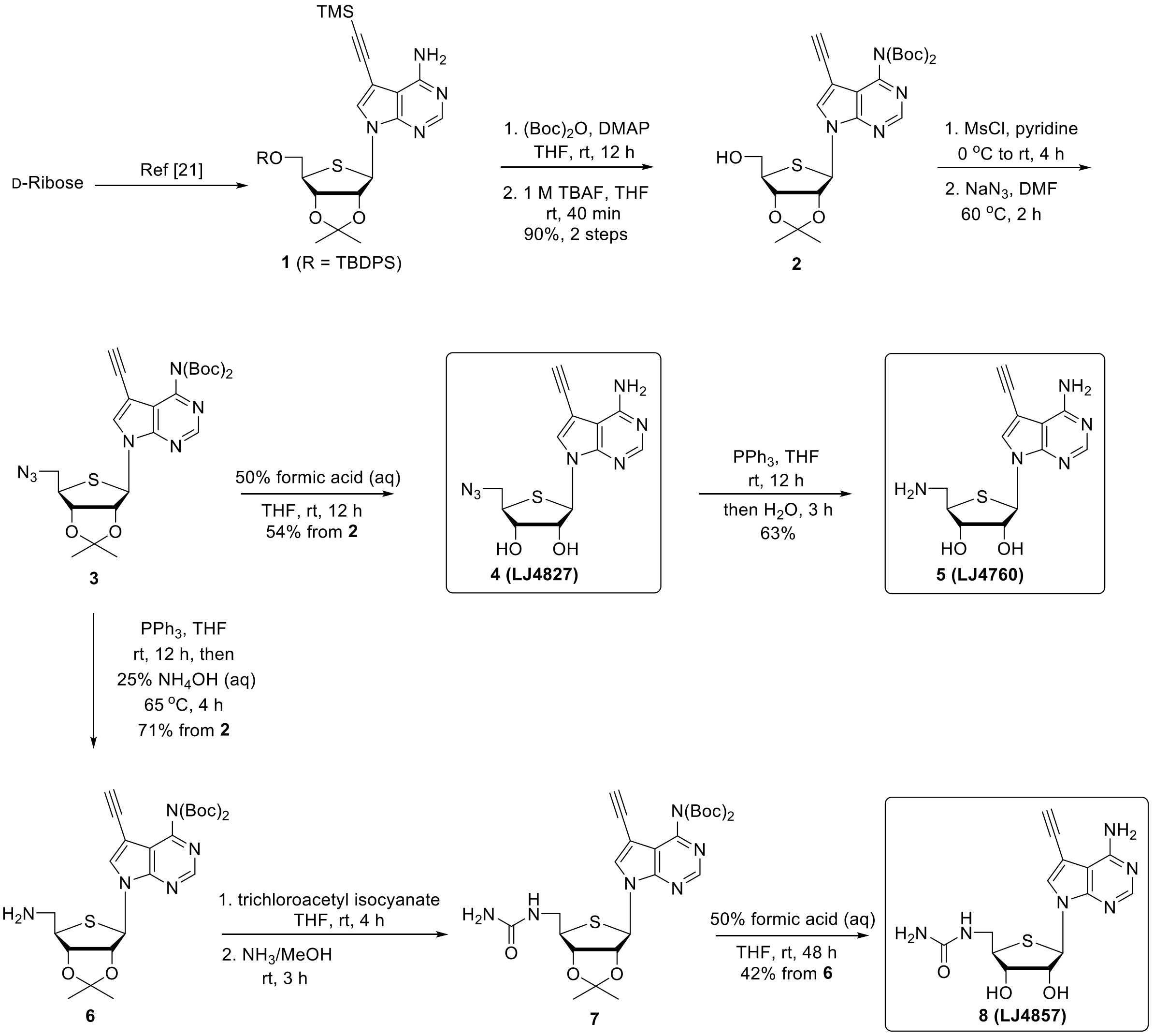

General Methods

Proton (^{1}H) and carbon (^{13}C) nuclear magnetic resonance spectra were recorded on a JEOL JNM-GCX (400/100 MHz) or Bruker AMX-500 (500/125 MHz) spectrometer in the solvent indicated. Chemical shifts are given in δ values, and are referenced to the solvent peak. Coupling constants (J) are reported in Hertz (Hz). High-resolution mass (HRMS) data were obtained on a Thermo LCQ XP instrument. UV spectra were recorded on a U-3000 made by Hitachi. Jasco III was used to measure optical rotations and $[\alpha]^{25}_D$ values are given in 10^{-1} deg

cm^2 g^{-1}. Melting points were recorded on a Barnstead electrothermal 9100 instrument and are uncorrected. The TLC spots analyzed under ultraviolet light at 254 nm were further visualized by *p*-anisaldehyde or phosphomolybdic acid stain solution. Column chromatography was performed on silica gel (Kieselgel 60, 70-230 mesh, Merck).

***tert*-Butyl (*tert*-butoxycarbonyl)(5-ethynyl-7-((3a*R*,4*R*,6*R*,6a*S*)-6-(hydroxymethyl)-2,2-dimethyltetrahydrothieno[3,4-*d*][1,3]dioxol-4-yl)-7*H*-pyrrolo[2,3-*d*]pyrimidin-4-yl)carbamate (2)**. To a solution of **1** [21] (0.85 g, 1.29 mmol) in THF (8.5 mL) was added DMAP (0.47 g, 3.88 mmol) followed by di-*tert*-butyl dicarbonate (1.77 mL, 7.76 mmol) at room temperature. After stirring at same temperature for 12 h, the volatiles were evaporated and the residue was partitioned between water (300 mL) and ethyl acetate (3 × 200 mL). The organic layer was separated, combined, dried over $MgSO_4$, filtered, and concentrated to give the crude compound.

This crude compound was dissolved in anhydrous THF (5.8 mL) and a solution of 1 M TBAF in THF (2.64 mL, 2.64 mmol) was added to it under nitrogen atmosphere and the resulting reaction mixture was stirred at room temperature for 40 min. The reaction was quenched with saturated aqueous NH_4Cl solution (5 mL) and extracted with ethyl acetate (3 × 150 mL). The combined organic layer was washed with brine (100 mL), dried over $MgSO_4$, filtered, and concentrated to give the residue which on purification afforded **2** (0.63 g, 90% in 2 steps) as sticky mass; silica gel column chromatography (CH_2Cl_2/MeOH, 49:1); $[\alpha]^{25}_D$ -74.61 (*c* 5.65, CH_3OH); UV (CH_3OH) λ_{max} 235.29 nm; ^1H NMR (CD_3OD, 400 MHz): δ 8.78 (s, 1H), 8.30 (s, 1H), 6.46 (d, J = 2.8 Hz, 1H), 5.21 (dd, J = 5.6, 3.2 Hz, 1H), 5.02 (dd, J = 5.2, 1.2 Hz, 1H), 3.79-3.74 (m, 3H), 3.67 (s, 1H), 1.61 (s, 3H), 1.34 (s, 21H); ^{13}C NMR (CD_3OD, 100 MHz): δ 152.1, 151.6, 151.4, 149.9, 133.1, 116.6, 111.9, 95.8, 89.3, 85.6, 83.7, 81.1, 74.3, 67.4, 63.7, 55.7, 26.7, 26.5, 24.1; HRMS (ESI-Q-TOF) *m*/*z* $[M + H]^+$ for $C_{26}H_{35}N_4O_7S$ calculated 547.2221, found 547.2214.

***tert*-Butyl (7-((3a*R*,4*R*,6*R*,6a*S*)-6-(azidomethyl)-2,2-dimethyltetrahydrothieno[3,4-*d*][1,3]dioxol-4-yl)-5-ethynyl-7*H*-pyrrolo[2,3-*d*]pyrimidin-4-yl)(*tert*-butoxycarbonyl) carbamate (3)**. Mesyl chloride (0.1 mL, 1.46 mmol) was added to a solution of **2** (0.40 g, 0.73 mmol) in anhydrous pyridine (15 mL/mmol) at 0 °C under nitrogen atmosphere and the reaction mixture was stirred at room temperature for 4 h. The volatiles were evaporated under vacuum and the residue was partitioned between water (220 mL) and ethyl acetate (3 × 160 mL). The separated organic layers were combined, dried over $MgSO_4$, filtered, and evaporated under vacuum below 25 °C water bath temperature. The crude mesylate was dissolved in anhydrous DMF (5.5 mL/mmol) and sodium azide (0.14 g, 2.16 mmol) was added portion wise at room temperature. The reaction mixture was transferred to preheated bath at 60 °C and stirred at the same temperature for 2 h. The solvent was evaporated under reduced pressure and the residue was partitioned between water (200 mL) and ethyl acetate (3 × 180 mL). The combined organic layers were dried over $MgSO_4$, filtered and concentrated to give crude **3** (R_f = 0.50, TLC eluent = hexane/ethyl acetate, 4:1) which was used as in the next step; HRMS (ESI-Q-TOF) *m*/*z* $[M + H]^+$ for $C_{26}H_{34}N_7O_6S$ calculated 572.2286, found 572.2277.

(2*R*,3*R*,4*S*,5*R*)-2-(4-amino-5-ethynyl-7*H*-pyrrolo[2,3-*d*]pyrimidin-7-yl)-5-(azidomethyl)tetrahydrothiophene-3,4-diol (4). To the crude intermediate **3** was added 50% aqueous formic acid solution (27 mL) and THF (5 mL). The resulting solution was stirred at room temperature for 12 h. Acidic solution was basified using a weakly basic anion-exchange resin (Dowex® 66 free base) and stirred for additional 3 h, filtered, and concentrated in vacuum. The residue was purified by silica gel column chromatography (CH_2Cl_2/MeOH, 24:1) to obtain **4** (0.13 g, 54% in 3 steps; 0.40 g scale of **2**) as white solid; mp 110-112 °C; $[\alpha]^{25}_D$ -30.23 (*c* 2.36, CH_3OH); UV (CH_3OH) λ_{max} 283.53 nm; IR ν_{max} strong band at 2169 cm^{-1}; ^1H NMR (DMSO-d_6, 500 MHz): δ 8.12 (s, 1H), 7.94 (s, 1H), 6.10 (d, J = 6.8 Hz, 1H), 5.59 (d, J = 6.2 Hz, 1H), 5.51 (d, J = 4.5 Hz, 1H), 4.52-4.48 (m, 1H), 4.30 (s, 1H), 4.09-4.07 (m, 1H),

3.86 (dd, J = 12.5, 6.9 Hz, 1H), 3.75 (dd, J = 12.5, 7.3 Hz, 1H), 3.38-3.34 (m, 1H); ^{13}C NMR (DMSO-d_6, 100 MHz): δ 157.4, 152.9, 149.9, 127.4, 102.2, 94.3, 83.2, 77.2, 76.8, 73.8, 61.3, 53.7, 50.1; HRMS (ESI-Q-TOF) m/z [M + H]$^+$ for $C_{13}H_{14}N_7O_2S$ calculated 332.0924, found 332.0923.

(2*R*,3*R*,4*S*,5*R*)-2-(4-amino-5-ethynyl-7*H*-pyrrolo[2,3-*d*]pyrimidin-7-yl)-5-(aminomethyl)tetrahydrothiophene-3,4-diol (5). To a solution of **4** (0.07 g, 0.21 mmol) in THF (5.6 mL) was added triphenylphosphine (0.11 g, 0.42 mmol) and the resulting solution was stirred at room temperature for 12 h. Water was added to the reaction mixture and stirred at room temperature for another 3 h. The volatiles were evaporated and the residue was purified by silica gel column chromatography (CH_2Cl_2/MeOH, 89:11) to afford **5** (0.04 g, 63%) as white solid; mp 125-127 °C; $[\alpha]^{25}_{D}$ -26.98 (c 0.56, CH_3OH); UV (CH_3OH) λ_{max} 279.41 nm; ^{1}H NMR (DMSO-d_6, 400 MHz): δ 8.12 (s, 1H), 7.94 (s, 1H), 6.07 (d, J = 6.7 Hz, 1H), 5.45 (d, J = 6.1 Hz, 1H), 4.45-4.41 (m, 1H), 4.30 (s, 1H), 4.12 (merged dd, J_1 = J_2 = 3.6 Hz, 1H), 3.26-3.21 (m, 1H), 3.0 (dd, J = 13.4, 6.1 Hz, 1H), 2.81 (dd, J = 13.4, 7.3 Hz, 1H); ^{13}C NMR (DMSO-d_6, 100 MHz): δ 157.4, 152.8, 149.9, 127.6, 102.2, 94.1, 83.1, 77.4, 77.3, 73.9, 61.1, 54.0, 45.4; HRMS (ESI-Q-TOF) m/z [M + H]$^+$ for $C_{13}H_{16}N_5O_2S$ calculated 306.1019, found 306.1031.

***tert*-Butyl (7-((3a*R*,4*R*,6*R*,6a*S*)-6-(aminomethyl)-2,2-dimethyltetrahydrothieno[3,4-*d*][1,3]dioxol-4-yl)-5-ethynyl-7*H*-pyrrolo[2,3-*d*]pyrimidin-4-yl)(*tert*-butoxycarbonyl)carbamate (6).** The crude **3** (1 equiv) was dissolved in anhydrous THF (27 mL/mmol) and to it was added triphenylphosphine (2 equiv) under nitrogen atmosphere. The reaction mixture was stirred at room temperature for 12 h. Ammonium hydroxide (25%, 1.8 mL/mmol) was added to the resulting solution and heated to 65 °C for 4 h. The volatiles were evaporated under vacuum and the residue was purified by silica gel column chromatography (CH_2Cl_2/MeOH, 19:1) to afford **6** (0.35 g, 71% in 3 steps; 0.50 g scale of **2**) as pale yellow sticky mass; $[\alpha]^{25}_{D}$ -27.61 (c 1.52, CH_3OH); UV (CH_3OH) λ_{max} 280.92 nm; ^{1}H NMR (CD_3OD,

400 MHz): δ 8.80 (s, 1H), 8.11 (s, 1H), 6.42 (d, J = 3.2 Hz, 1H), 5.32 (dd, J = 5.6, 2.8 Hz, 1H), 5.01 (dd, J = 6.0, 2.8 Hz, 1H), 3.70-3.66 (s merged with m, 2H), 3.05-3.00 (m, 1H), 2.88-2.85 (m, 1H), 1.60 (s, 3H), 1.34 (s, 21H); HRMS (ESI-Q-TOF) *m*/*z* $[M + H]^+$ for $C_{26}H_{36}N_5O_6S$ calculated 546.2381, found 546.2372.

***tert*-Butyl (*tert*-butoxycarbonyl)(7-((3a*R*,4*R*,6*R*,6a*S*)-2,2-dimethyl-6-(ureidomethyl)tetrahydrothieno[3,4-*d*][1,3]dioxol-4-yl)-5-ethynyl-7*H*-pyrrolo[2,3-*d*]pyrimidin-4-yl)carbamate (7).** Trichloroacetyl isocyanate (0.075 g, 0.40 mmol) was added dropwise to a stirred solution of **6** (0.22 g, 0.40 mmol) in anhydrous THF (6.6 mL) and the reaction mixture was stirred at room temperature for 4 h. Reaction was quenched with water (25 mL) and extracted with ethyl acetate (3 × 60 mL). Organics were combined, dried, filtered and concentrated to give crude which was utilized for the next step without any purification. To the crude was added NH_3/*tert*-BuOH (5 mL) and stirred at room temperature for 3 h. Volatiles were evaporated under vacuum and the crude residue **7** (R_f = 0.60, TLC eluent = CH_2Cl_2/MeOH, 9:1) was subjected to the next reaction; HRMS (ESI-Q-TOF) *m*/*z* $[M + H]^+$ for $C_{27}H_{37}N_6O_7S$ calculated 589.2439, found 589.2447.

1-(((2*R*,3*S*,4*R*,5*R*)-5-(4-amino-5-ethynyl-7*H*-pyrrolo[2,3-*d*]pyrimidin-7-yl)-3,4-dihydroxytetrahydrothiophen-2-yl)methyl)urea (8). The crude **7** was converted to **8** (0.06 g, 42% in 3 steps) as white solid, by following the procedure described for **4**; silica gel column chromatography (CH_2Cl_2/MeOH, 89:11); mp 114-116 °C; $[\alpha]^{25}_D$ -21.79 (*c* 0.06, CH_3OH); UV (CH_3OH) λ_{max} 281.18 nm; ^1H NMR (CD_3OD, 400 MHz): δ 8.11 (s, 1H), 7.79 (s, 1H), 6.16 (d, J = 6.0 Hz, 1H), 4.47 (dd, J = 5.6, 3.6 Hz, 1H), 4.16-4.14 (m, 1H), 3.73 (s, 1H), 3.60-3.52 (m, 1H), 3.50-3.41 (m, 2H); ^{13}C NMR (DMSO-d_6, 125 MHz): δ 158.5, 157.4, 152.8, 150.0, 127.4, 102.2, 94.2, 83.0, 77.3, 77.1, 73.9, 61.0, 51.8; HRMS (ESI-Q-TOF) *m*/*z* $[M + H]^+$ for $C_{14}H_{17}N_6O_3S$ calculated 349.1077, found 349.1092.

2. Biological Assay

Cell culture

Hela, A549 and hMSCs cell lines were maintained in Dulbecco's modified Eagle Medium (DMEM). DMEM were supplemented with 10% (v/v) fetal bovine serum, gentamicin (50 μg/mL) at 37 °C in a humidified atmosphere of 5% CO_2 in the air.

Annexin-V & 7-AAD staining

To determine the population of dead cells, Annexin-V & 7-AAD staining was carried out in accordance with the manufacturer's instructions (559763, BD Pharmingen). Results were analyzed using a Becton-Dickinson FACS Calibur-1 with the Cell-QUEST software.

PI staining & Cell cycle profiling

PI staining to determine cell cycle distribution (Sub G1, G1, S and G2/M) based on DNA content. The stained cells were analyzed using Becton-Dickinson FACS Calibur-1 and Modfit 4.1 cell cycle analysis software.

Cell synchronization

Using double thymidine block (DTB), FUCCI-HeLa cells were synchronized at the G1/S boundary. In detail, cells were treated with 2.5 mM thymidine for 16 h and were released back with normal medium for 8 h. After second thymidine treatment with 2.5 mM thymidine for an additional 16 h, cells were arrested at G1/S boundary. Cells were live monitored with the JULI-stage live image machine (NanoEntek).

Time-lapse imaging and compensation

Time-lapse images were acquired at constant time points (Bright field (BF), GFP, and RFP 3-channel. LED power: 1, Bright: 4, RFP is 7. Exposure: 400 ms, BF is 70 ms. Images captured with auto-focusing in all channels and cycles) using JULI-stage software (NanoEntek), and contrast of both green and red fluorescence compensated using batch editor of photoscape software (Auto contrast: middle. Contrast: low, +5. Erode). Attached cell counter of JULI-stat

(NanoEntek) was used as analyzing software (intensity of min: 10, max: 255, counted for each pixel) [1].

Clonogenic assay

A549 and Hela cells were seeded in 6 well plate at cell concentrations estimated to yield 20–100 colonies/well. After 24 hours of culture, cells were pretreated according to appropriate concentrations. Cells were cultured for 10–14 d, and colonies were counted using Image J.

Drug combination analysis

CompuSyn software (version 1.0; T. C. Chou and N. Martin, Memorial Sloan-Kettering Cancer Centre, New York) was used to calculate the combination index (CI)

Adenosine kinase activity assay

hAdK activity was measured using a commercial kit to assess the nucleoside derivatives for inhibitory action (ref. no. K0507-02, NovoCib, Lyon, France). hAdK activity was assayed by measuring specific absorption at 340 nm at 5 min time intervals using a Spectromax plate reader (Molecular Devices, CA, USA)

HASPIN kinase activity assay

The Haspin Kinase Enzyme System (ref. no. VA7468, Promega, Korea) with using ADP-GLO kinase assay (ref. no. VA6930, Promega, Korea) measured ADP formed from a kinase reaction; ADP is converted into ATP, which is a substrate in a reaction catalyzed by Ultra-Glo™ Luciferase that produces light. The luminescent signal positively correlates with ADP amount and kinase activity. The luminescent signal was assayed by a Spectromax plate reader (Molecular Devices, CA, USA)

Immunoblotting and immunofluorescence

Cells were lysed with tissue lysis buffer supplemented with 0.2 mM sodium vanadate and 1 mM protease inhibitor cocktail (Roche, Basel, Switzerland), and for immunoblotting. For immunofluorescence, cell was fixed with 4% PFA for 10 min at RT followed by

permeabilization with 0.1% Triton X-100 for 2 min and blocking with 3% BSA for 1 hr at RT. Antibodies for Cyclin B1 (#sc-245) and β-actin (#sc-47778), were purchased from Santa Cruz Biotechnology. Antibodies for pH2AX (#9718S), pH3ser10 (#9710S), CENP-F(#58982S), and cleaved caspase-3 (#9664S) were purchased from Cell Signaling Biotechnology. Antibodies for pH3T3(ab222775) and AURKB (ab3609) were purchased from Abcam. HASPIN (NBP1-26626) was purchased from Novus Biologicals.

In Vivo Tumor Xenograft Model

All animal experiments were conducted according to the guidelines approved by the Seoul National University Institutional Animal Care and Use Committee (IACUC permission number SNU-220217-1). Balb/c-nu mice (male, 4-weeks-old; OrientBio, Seoul, Korea) were allowed one-week acclimation prior to the experiment. A549 cells (4.5×10^6 cells in 200 µL PBS) were injected subcutaneously into the flanks of mice, and tumors were allowed to grow for 14 days until their volume reached approximately 70 mm^3. The mice were randomly divided into four groups for vehicle control and treatment groups (n = 5); vehicle control (DMSO: cremophor: normal saline = 1:1:18), LJ4827 (1 mg/kg body weight), BI2536 (10 mg/kg body weight), or a combination of LJ4827 (1 mg/kg body weight) and BI2536(10 mg/kg body weight). LJ4827 compound was dissolved in vehicle (DMSO: cremophor: normal saline = 1:1:18) and BI2536 compound was dissolved in vehicle (polyethyleneglycol-400: normal saline = 1:4). LJ4827 and vehicle control (DMSO: cremophor: normal saline = 1:1:18) were intraperitoneally administered three times per week. BI2536 was intraperitoneally administered two times per week, and a combination of LJ4827 and BI2536 were administered each three times per week and two times per week for 23 days. The body weight and tumor volume were measured every 2-3 days. The tumor volume was measured using a digital slide caliper according to the following formula: tumor volume (mm^3) = 0.52 × (width × length × height).

Statistical analysis

The quantitative data are expressed as the mean values ± standard deviation (SD). Student's unpaired t-tests for two groups or one-way ANOVA following Tukey mulpile comparison, was performed to analyze the statistical significance of each response variable using the PRISM. p values less than 0.05 were considered statistically significant (*, $p < 0.05$, **, $p < 0.01$, ***, $p < 0.001$, ****, $p < 0.0001$ and n.s for not significant).

3. Molecular Docking Studies

Cloning, expression, and purification of HASPIN for structure determination

The kinase domain of human HASPIN (residue 452 – 798) was cloned into the expression vector pET-21a(+) (Novagen, Madison, WI, USA). The genomic DNA of HASPIN was provided from the Korea Human Gene Bank, Medical Genomics Research center, KRIBB, Korea. The plasmid that contains the kinase domain of human HASPIN was transformed into *Escherichia coli* strain Rosetta^{TM} 2(DE3) pLysS (Novagen, Madison, WI, USA). The transformed cells were grown at 37°C until OD_{600} reached 0.8 and induced with 0.5 mM isopropyl β-D-1-thiogalactopyranoside (IPTG). After incubation for additional 18 h at 20°C, the cells were harvested and resuspended using lysis buffer [20 mM Tris-HCl, pH 7.5, 500 mM NaCl, 35 mM imidazole, and 1 mM phenylmethanesulfonylfluoride]. For the affinity chromatography, 5 mL HiTrap^{TM} chelating HP column (GE Healthcare, Chicago, IL, USA) charged with Ni^{2+} was used. The column was equilibrated with buffer A [20 mM Tris-HCl, pH 7.5, 500 mM NaCl, and 35 mM imidazole] and eluted with buffer B [20 mM Tris-HCl, pH 7.5, 500 mM NaCl, and 1 M imidazole]. The eluent was loaded into HiLoad 16/600 Superdex75^{TM} pg column (GE Healthcare, Chicago, IL, USA) that was pre-equilibrated with buffer C [20 mM Tris, pH 7.5, 250 mM NaCl, 10% glycerol, and 1 mM 1,4-dithiothreitol]. The purified HASPIN protein was finally concentrated to 5 mg/mL for crystallization experiments.

Crystallization, data collection, and structure determination of HASPIN in complex with LJ4827 and with LJ4760

LJ4827 and LJ4760 were respectively added to the purified HASPIN protein with molar ratio of 1:3 and were co-crystallized by the sitting-drop vapor diffusion method at 22°C. Diffraction-quality crystals were obtained at the reservoir containing 23.75% PEG 4000, 0.2 M ammonium sulfate, and 0.1 M sodium acetate at pH 4.6 for LJ4827, and 23.75% PEG4000, 0.325 M ammonium sulfate, and 0.1 M sodium acetate at pH 4.6 for LJ4760. Crystals were

cryoprotected in the crystallization solution supplemented with 30% glycerol and flash-frozen into liquid nitrogen. X-ray diffraction data were collected at the Eiger 9M detector (Dectris Ltd., Baden, Switzerland) at the beamline 5C of the Pohang Light Source, Korea. HKL2000 program suite was used for raw data indexing and scaling (Otwinowski and Minor, 1997). The structures of the kinase domain of HASPIN in complex with LJ4827 and with LJ4760 were solved by the molecular replacement method using the HASPIN structure with PDB ID 2WB8 (Villa et al., 2009) as a reference model in the PHENIX Phaser-MR program (Liebschner et al., 2019). The structures were further refined by Coot in the CCP4i program suite (Emsley et al., 2010) and phenix.refine in PHENIX (Liebschner et al., 2019).

Docking studies

AutoDock Vina (Trott and Olson, 2010) was used for docking studies. The HASPIN structure in complex with LJ4827 was used for the control docking experiment and the grid box sized with 10×10×10 points was centered at the LJ4827 binding site with 1.0 Å spacing. LJ4760, 5ITU, CHR6494 were used as ligands for docking and the structures of ligands were obtained using PRODRG (Schuttelkopf and van Aalten, 2004).

4. Computational Analysis

Generation and preprocessing of RNA sequencing data

Total RNA was isolated from HeLa cells before and after treatment with vehicle, LJ 4827, or 5ITU in duplicates using Trizol according to the manufacturer's instructions. For library construction, we used the TruSeq Stranded mRNA Library Prep Kit (Illumina, San Diego, CA). Briefly, the strand-specific protocol included the following steps: (1) strand cDNA synthesis, (2) strand synthesis using dUTPs instead of dTTPs, (3) end repair, A-tailing, and adaptor ligation, and (4) PCR amplification. Each library was then diluted to 8 pM for 76 cycles of paired-read sequencing (2 × 75 bp) on an Illumina NextSeq 500 following the manufacturer's recommended protocol. Read quality was assessed using FastQC (v) and poor-quality bases (Phred score < 20) were eliminated using TrimGalore (v0.6.6). Trimmed reads were aligned to the human reference genome (GRCh38) using the STAR aligner (v2.7.9a) with default parameters. Gene-level expression values such as transcripts per million (TPM) and read counts were calculated using RSEM (v1.3.3.) with human gene annotation (GRCh38.84). FASTQ format files, gene-level count data, and TPM of all samples are available in the Gene Expression Omnibus. Among the xxx genes, xxx protein-coding genes were utilized for subsequent analysis. Differential gene expression analysis was performed using the 'DESeq2' package (v3.15) in R (v4.2.1). The FASTQ files and processed data (read count) are available in the Gene Expression Omnibus (GEO GSE213563).

CMap analysis to infer MoA

CMap provides a web-based tool for searching for compounds that give rise to similar or dissimilar expression signatures to an input signature, referred to as a set of differentially expressed genes. It contains an extensive catalog of transcriptome profiles for 9 core human cell lines (A375, A549, HA1E, HCC515, HEPG2, HT29, MCF7, PC3 and VCAP) before and after treatment with each of 29,679 small molecules. To infer the MoA of LJ 4827, we queried

CMap reference database for differentially expressed genes induced by LJ 4827 treatment (DEGs, top or bottom 150 genes selected based on Wald statistic values of DESeq2 results) and obtained a list of compounds that induce similar expression signatures to LJ 4827 (normalized connectivity score > 2).

Chemical similarity of compounds

Simplified Molecular Input Line Entry System (SMILES) of 18 top-scoring compounds and LJ 4827 were obtained from ChEMBL (https://www.ebi.ac.uk/chembl/ Accessed August, 2022). Structural similarity between compounds was determined by Tanimoto coefficients calculated using RDKit (v2022.03.1) with default settings in Python3 (v3.9).

Functional enrichment analysis

Quantitative changes in gene expression levels between groups (treatment vs vehicle, tumor vs normal) were estimated by using the 'DESeq2' package in R. Differentially expressed genes were selected with cutoffs of false discovery rate (FDR) adjusted P-value < 0.01 and |fold change| ≥ 2. Over-representation analysis of Gene Ontology (GO) among DEGs of LJ4827 and 5ITU was performed using the R package topGO. Gene set enrichment analysis (GSEA) was performed with the REACTOME database based on a list of genes ranked by Wald statistic from DESeq2 result via the 'msigdbr' (v7.5.1) and 'fgsea' (v1.22.0) package in R.

The Cancer Genome Atlas (TCGA) transcriptome data analysis

Transcriptome (TPM) and clinical information of 9563 cancer patients in Pan-Cancer Atlas were obtained from the UCSC Xena Functional Genomics Explorer (https://xenabrowser.net/ Accessed August, 2022) and cBioPortal (https://www.cbioportal.org/ Accessed August, 2022), respectively. Active mitosis signature enrichment score (AMSES) of each patient was computed by single-sample GSEA (ssGSEA) analysis using the active mitosis signature. The ssGSEA was conducted through R packages 'fgsea' based on patient-wise z-transformed TPM values of all genes. Survival analysis was conducted to test the difference in the survival rate

in patients between two groups, high and low, determined based on GSG2 expression level or Active mitosis signatures score. The hazard ratio (HR) and P-value were estimated from Cox proportional hazards regression analysis and the log-rank test by using the package 'survival' (v3.4-0) in R. The synthetic lethal (SL) partner of CSG2 was explored within the active mitosis signature by using tumor transcriptome data of TCGA LUAD patients (N=508). Among the signature, genes encoding kinases were considered candidates for SL partners due to their druggability. Patients were divided into GSG2-high and GSG2-low groups based on *GSG2* expression. Association between kinase gene expression and overall survival rates in the GSG2-low group or GSG2-high group were estimated using a Cox regression analysis.

Supplementary Figures

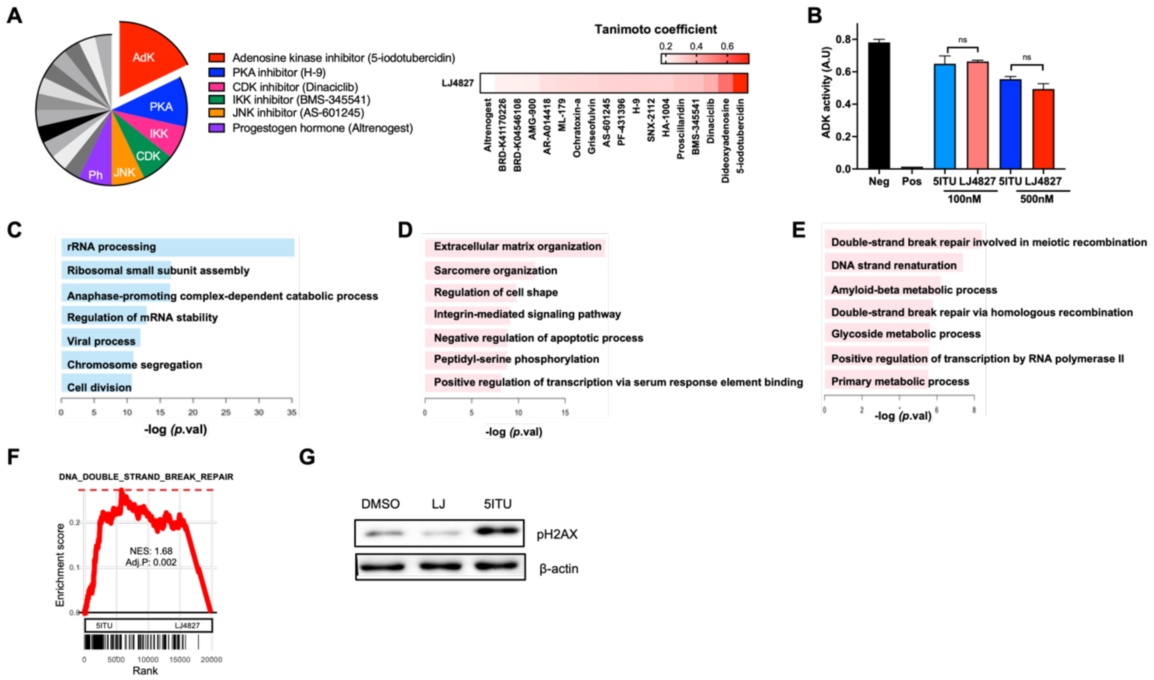

Figure. S1 (A) Frequency of matched compound’s mode of action in query and Tanimoto coefficients of compounds predicted in query. (B) Adenosine kinase activity after LJ4827 or 5ITU treatment. (C) Gene ontology analysis of downregulated genes by both LJ4827 and 5ITU treatment. (D) Gene ontology analysis of downregulated genes by only LJ4827 treatment. (E) Gene ontology analysis of downregulated genes by only 5ITU treatment. (F) GSEA plot of the enrichment of the “DNA_double_strand_breeak_repair” signature in the 5ITU-treated group in comparison with the LJ4827-treated group. (G) Immunoblotting for pH2AX at 24 h after treatment of LJ or 5ITU in HeLa.

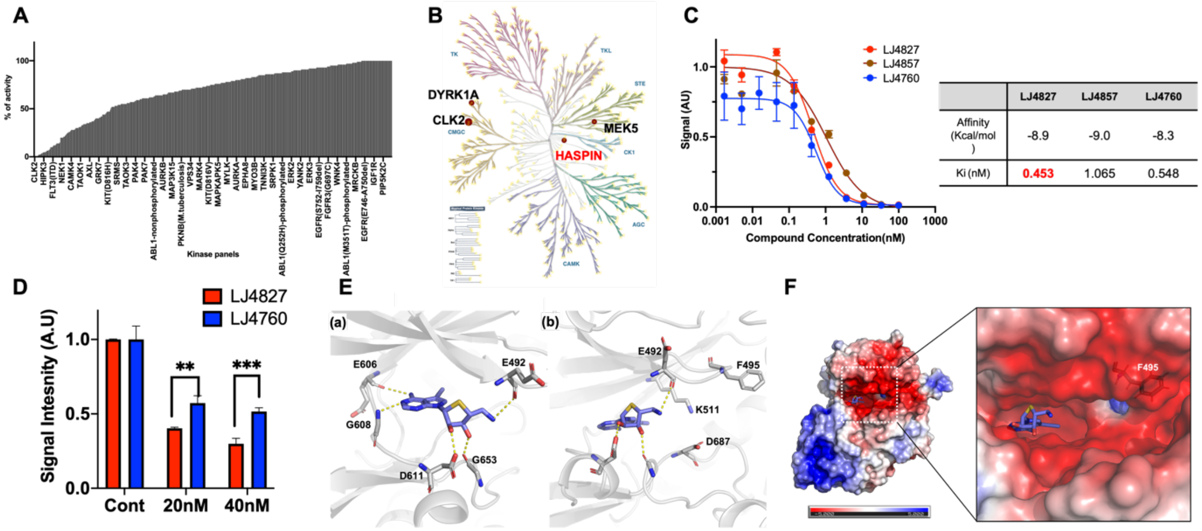

Figure. S2 (A) graphical presentation of Kinomescan profiling of 100 nM LJ4827on total 468 kinases. (B) KinMAP of LJ4827 under 1% of control. (C) Binding constant values of LJ4827, LJ4857, and LJ4760 for HASPIN. (D) in vitro kinase assay of HASPIN with the indicated concentrations of LJ4827 and LJ4760 using Histone H3 as a substrate. (E) Interaction between the HASPIN hinge region (a) and interaction between HASPIN and the amino moiety of LJ4760 (b). (F) Surface electrostatic potential map of HASPIN in complex with LJ4760.

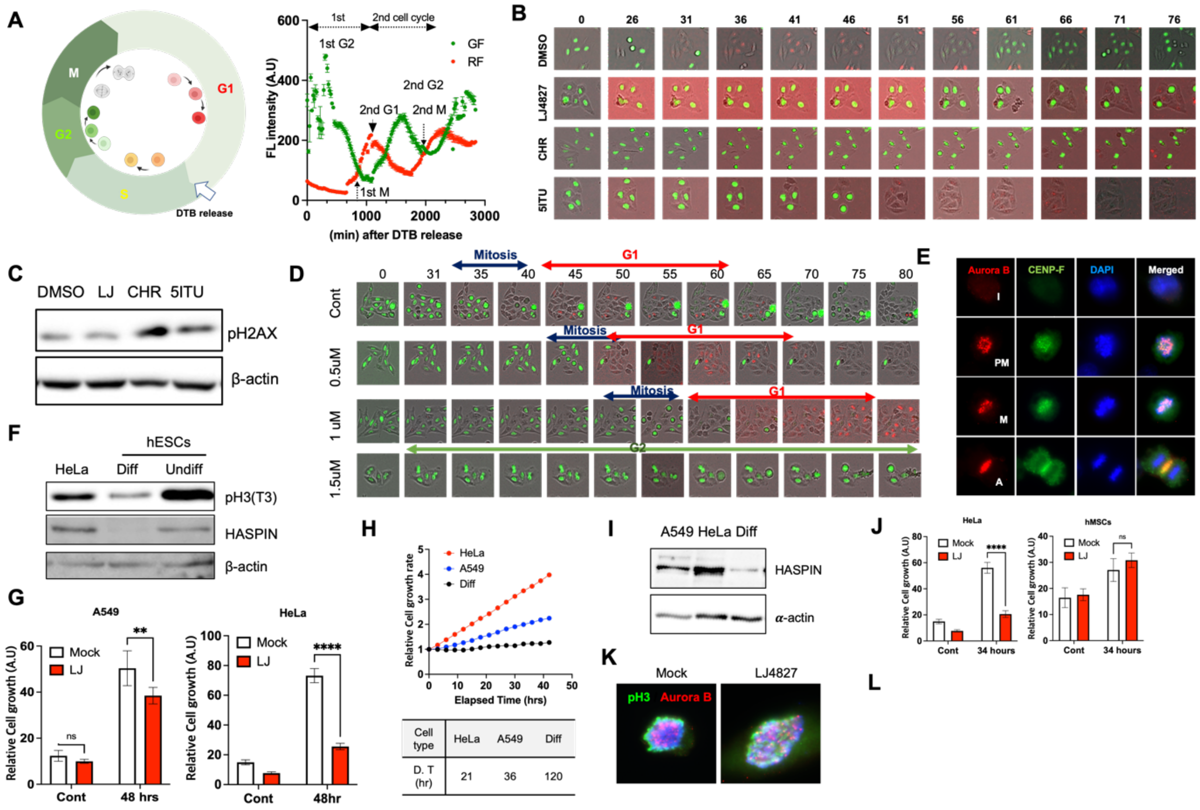

Figure. S3 (A) Scheme of the FUCCI system and temporal intensity profiles of green or red fluorescence from FUCCI-HeLa at indicated time after release from double thymidine block. (B) Time-lapse images of cell cycle after HASPIN inhibitors treatment, 1 frame = 20-minute. (C) Immunoblotting analysis for pH2AX on A549 cells at 24 h after 500 nM treatment of each compound. (D) Time-lapse images of cell cycle after LJ4827 concentration-dependent treatment. (E) Immunofluorescent images of Aurora B and CENP-F in HeLa (I: interphase, PM: prometaphase, M: metaphase and A: Anaphase) (F) Immunoblotting analysis for HASPIN and phospho-Histone H3 on HeLa, differentiated cells from hESCs and undifferentiated hESC. (G) Relative cell growth rate at 48 h after LJ4827 treatment on A549 and HeLa. (H) Relative cell growth rate on HeLa, A549, and differentiated cells from hESCs, estimated doubling times were represented on bottom tables. (I) Immunoblotting for HASPIN in A549, HeLa and Diff. (J) Relative cell growth rate after LJ4827 treatment on HeLa and hMSCs. (K) Immunofluorescent images of phospho-Histone H3 [serine 10, pH3(S10)] (green) and Aurora

B (red) in hMSCs in the absence or presence of LJ4827. (L) Graphical presentation of the body weight after injection of indicated dose of LJ4827

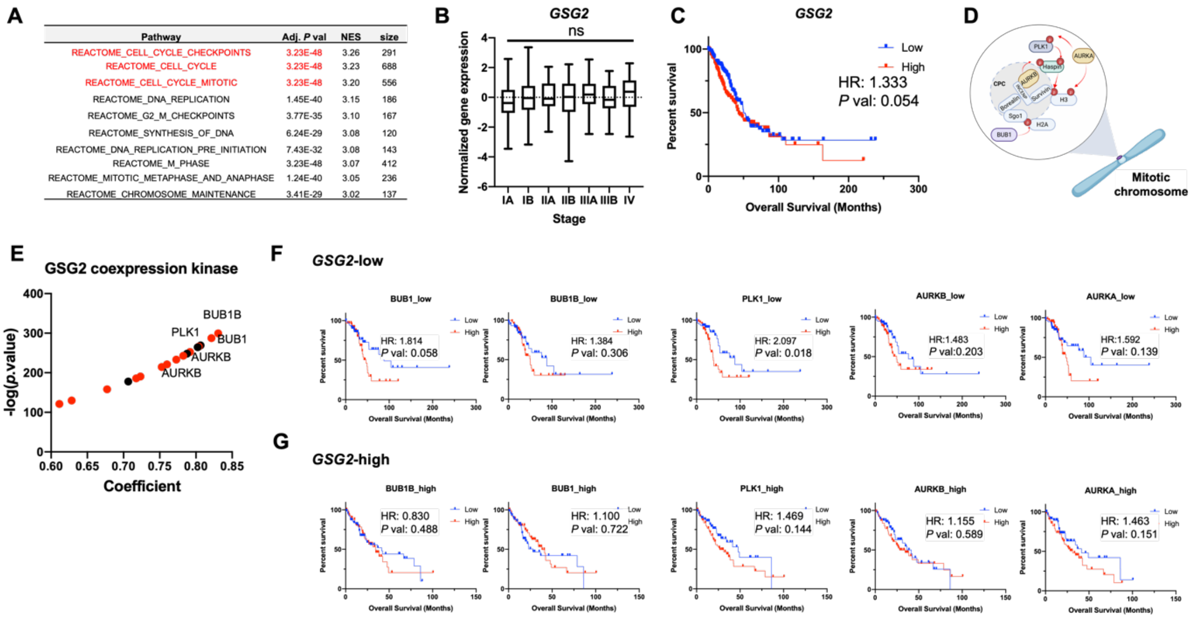

Figure. S4 (A) List of enriched REACTOME genesets in tumor group. (B) GSG2 expression levels by cancer stage. (C) Kaplan-Meier survival curves of overall survival by GSG2 expression. (D) Graphical presentation of selected kinases in CPC regulation at the mitotic chromosome. (E) Graph of genes highly correlated to GSG2 expression. (F and G) Kaplan-Meier plot for overall survival with BUB1, BUB1B, PLK1 and AURKB expression in GSG2 low (F) and high (G) patients

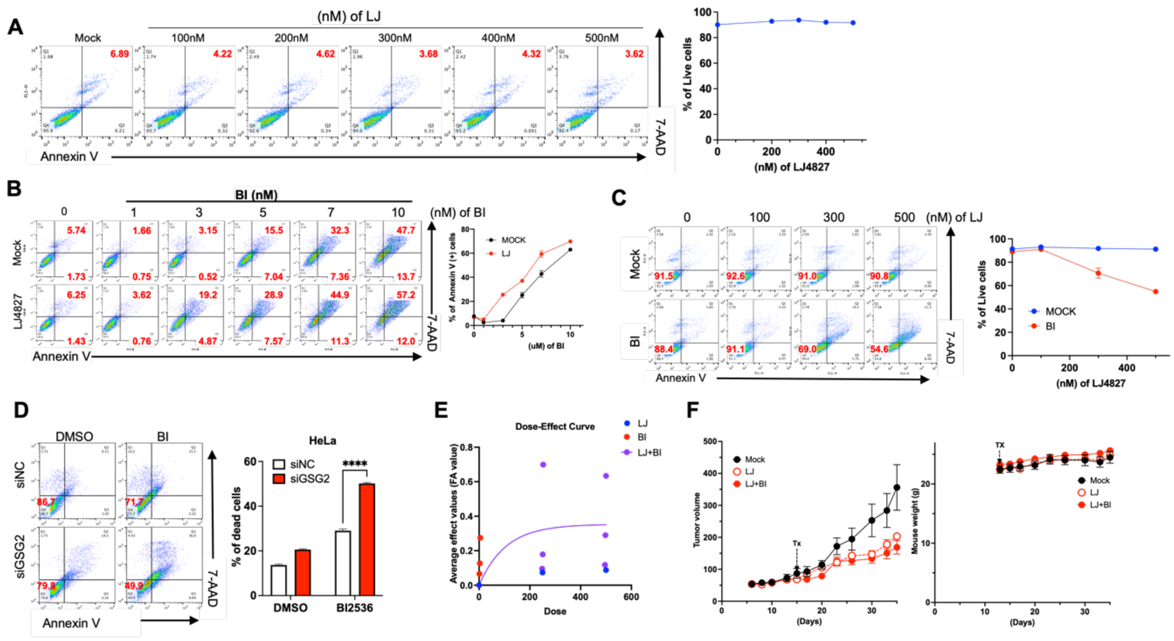

Figure. S5 (A) Flow cytometry for Annexin V and 7-AAD at 48 h after indicated dose of LJ4827 (left), graphical presentation of dual positive population with Annexin V and 7-AAD staining (right). (B) Flow cytometry for Annexin V and 7-AAD at 48 h after treatment of indicated dose of BI2536 (BI) in the absence or presence of LJ4827 (left), graphical presentation of annexin V positive cells (right). (C) Flow cytometry for Annexin V and 7-AAD at 48 h after indicated dose of LJ4827 concentration with and without BI2536 (left), quantitative measurement of Annexin V and 7-AAD both negative cells (right). (D) Flow cytometry for Annexin V and 7-AAD at 48 h after BI2536 treatment with siRNA (Negative control: siNC and GSG2: siGSG2), graphical presentation of annexin V and 7AAD negative cells (right). (E) Dose effect curve of six data points of LJ4827 and BI combination, data from CompuSyn software. (F) Tumor volume of tumor-bearing mice after the indicated treatment (left) and changes in body weight of tumor-bearing mice after treatment (right), Tx: time at compound treatment

Movie S1 (A) Timelapse video of cell cycle after vehicle treatment, (B) Timelapse video of cell cycle after LJ4827 treatment, (C) Timelapse video of cell cycle after CHR6494 treatment, (D) Timelapse video of cell cycle after 5ITU treatment

Movie S2 (A) Timelapse video of cell cycle after vehicle treatment, (B) Timelapse video of cell cycle after LJ4827 (0.5uM) treatment, (C) Timelapse video of cell cycle after LJ4827 (1uM) treatment, (D) Timelapse video of cell cycle after LJ4827 (1.5uM) treatment

Reference

(1) Go, Y. H.; Lee, H. J.; Kong, H. J.; Jeong, H. C.; Lee, D. Y.; Hong, S. K.; Sung, S. H.; Kwon, O. S.; Cha, H. J. Screening of cytotoxic or cytostatic flavonoids with quantitative Fluorescent Ubiquitination-based Cell Cycle Indicator-based cell cycle assay. *R Soc Open Sci* **2018**, *5* (12), 181303. DOI: 10.1098/rsos.181303.
